# Wheat (*Triticum aestivum*) chromosome 6D harbours the broad spectrum common bunt resistance gene *Bt11*

**DOI:** 10.1007/s00122-023-04452-5

**Published:** 2023-09-07

**Authors:** Magdalena Lunzer, Maria Buerstmayr, Heinrich Grausgruber, Almuth Elise Müllner, Iris Fallbacher, Hermann Buerstmayr

**Affiliations:** 1 Institute of Biotechnology in Plant Production, University of Natural Resources and Life Sciences, Konrad-Lorenz-Strasse 20, Tulln, Vienna, 3430 Austria; 2 Institute of Plant Breeding, University of Natural Resources and Life Sciences, Konrad-Lorenz-Strasse 24, Tulln, Vienna, 3430 Austria; 3 Present Address: Saatzucht Donau GesmbH & CoKG, Saatzuchtstrasse 11, Probstdorf, 2301 Austria; 4 Present Address: Österreichische Rübensamenzucht Ges.m.b.H, Josef-Reither-Straße 21-23, Tulln, 3430 Austria

## Abstract

**Key message:**

A major QTL on chromosome 6DL corresponding to bunt resistance gene *Bt11* was identified in four mapping populations generated through crosses with *Bt11*-carriers PI 166910 and M822123.

**Abstract:**

Common bunt in wheat has witnessed a renaissance with the rise of organic agriculture that began in the 1980s. The abandonment of systemic fungicides in organic farming, together with a lack of resistant cultivars, has led to wide-spread problems due to common bunt infections. Knowledge about genetic sources for resistance is still scarce and only few of the known bunt resistance factors are currently used in breeding. We therefore aimed to map the resistance factor harboured by the Turkish landrace PI 166910, which is the resistance donor for the *Bt11* bunt differential line. Four mapping populations (MPs) with 96–132 recombinant inbred lines (RILs) were phenotyped for common bunt resistance over 2, 3 or 4 years with one or two local bunt populations and genotyped with the 25K SNP array. A major bunt resistance locus on the distal end of chromosome 6D designated *QBt.ifa-6DL* was identified in all MPs and experiments. Additional QTL contributing to resistance were detected on chromosomes 4B, 1A, 1B, 2A and 7B. *QBt.ifa-6DL* mapped to a region overlapping with the *Bt9*-locus identified in previous studies, but results indicate that *QBt.ifa-6DL* is different from *Bt9* and convincing evidence from haplotype comparisons suggests that it represents the *Bt11* resistance allele. Markers for the distal region of chromosome 6D between 492.6 and 495.2 Mbp can be used to select for *QBt.ifa-6DL*. This resistance factor confers high and stable resistance against common bunt and should be integrated into organic and low-input wheat breeding programs.

**Supplementary Information:**

The online version contains supplementary material available at 10.1007/s00122-023-04452-5.

## Introduction

Organic farming aims to reduce problems in modern agriculture like soil erosion, soil depletion or decreasing diversity in crop plants (Kuepper 2010) but at the same time faces almost forgotten challenges which endanger crop yields. One prominent example of such a challenge is common bunt (CB) in wheat, caused by the fungal pathogens *Tilletia caries* (D.C.) Tul. & C. Tul. (*Tilletia tritici* (Bjerk.) G. Winter) and *T. laevis* J.G. Kühn (*T. foetida* (Wallr.) Liro). Until the first half of the nineteenth century, common bunt was among the most devastating wheat diseases destroying whole fields and causing so-called “black harvests,” e.g. in the Pacific North West of the U.S.A (Bruehl 1990; Matanguihan et al. 2011). With the introduction of highly efficient systemic fungicide treatments during the 1950s, this seed-borne disease was not given a lot of attention in wheat breeding programs any more (Line 1993). For organic agriculture, these seed treatments are not an option and farmers are faced with challenges due to a lack of breeding activities for resistance to common bunt over many decades (Saari and Mamluk 1996). This negligence of common bunt results in the availability of only few cultivars providing complete or at least partial resistance against the disease, especially in Europe and the U.S.A (Goates and Bockelman 2012; Liatukas and Ruzgas 2008). The (re-)consideration of common bunt resistance as a high priority goal for breeding is in fact beneficial for both conventional and organic farming. The deployment of resistant cultivars is the most economically efficient and at the same time environmentally friendly way of managing bunt diseases and provides advantages for conventional and low-input farming systems (Matanguihan et al. 2011; Saari and Mamluk 1996).

Already very low infection levels of 0.05% of grain weight can lead to quality reduction (Martens et al. 1984) and especially to a build-up of disease incidence over the following years if contaminated, untreated grain is used for sowing. Therefore, control measures need to have an efficiency of over 99% to provide protection (Borgen and Davanlou 2000). Apart from quality loss, infestation with common bunt also leads to yield losses in the same quantity as disease incidence since the wheat grains get replaced by fungal teliospores, resulting in so-called bunt balls or *sori* (Cherewick 1953; Hoffman 1982). Yield losses due to common bunt infections in wheat producing states of the U.S.A and Ontario, Canada, were estimated to amount to 2,215,441 bushels (30,294.3 tons) in 2021 and 519,051 bushels (14,126.2 tons) in 2022 (source: Wheat Disease Loss Calculator by the Crop Protection Network, available via https://loss.cropprotectionnetwork.org/crops/wheat-diseases [accessed 2023-07-19]). The disease’s common name “stinking smut” is derived from a strong smell of rotten fish caused by trimethylamines produced in the fungal spores (Chen et al. 2016; Matanguihan et al. 2011).

Resistance against common bunt has long been regarded as being of mainly qualitative genetic nature and based on the gene-for-gene concept of matching virulence and avirulence genes (Flor 1956; Goates 1996; Goates and Bockelman 2012; Hoffman and Metzger 1976). In recent years, though, also quantitative trait loci conferring a more complex and quantitative type of resistance have been detected (Bhatta et al. 2018; Mourad et al. 2018; Muellner et al. 2021). A bunt differential set consisting of wheat accessions putatively monogenic for bunt resistance genes *Bt1*–*Bt15* and *BtP* developed by Hoffman and Metzger (1976) and extended by Goates (2012) has been widely used for gene postulation and virulence monitoring of bunt populations by research groups around the world (Gordon et al. 2020; Liatukas and Ruzgas 2008; Muellner et al. 2020). Distinct patterns of virulence/avirulence against the individual *Bt* resistance genes present in the differential lines can be observed for bunt races from different origins. Blazkova and Bartos (2002) tested bunt races from several European countries and Syria and found that the only differential lines affected by none of these races were those carrying *Bt8*, *Bt11* and *Bt12*. These findings are in line with previous studies that could also not detect bunt races virulent to one of these three resistance factors (Goates 1996; Hoffman and Metzger 1976; Metzger and Hoffman 1978). In recent years, virulence of common bunt against both *Bt9* and *Bt10* has been detected in several European countries (Bengtsson et al. 2023; Dumalasová 2021; Ritzer et al. 2022). While this is still a rather rare phenomenon, virulence against *Bt2* and *Bt7* has been reported in a wide range of environments (Cadot et al. 2021; Ehn et al. 2022; Goates and Bockelman 2012). Recent studies conducted in Sweden and the U.S.A additionally identified common bunt races virulent to *Bt1*, *Bt3*, *Bt4*, *Bt8*, *Bt13* and *BtP* (Bengtsson et al. 2023; Joshi et al. 2023).

Out of the 17 *Bt*-genes postulated to date, only *Bt9*, *Bt10* and *Bt12* have been mapped and have linked markers available facilitating their use in practical breeding programs. The first bunt resistance gene to be mapped was *Bt10* which is located on the short arm of chromosome 6D (Laroche et al. 2000; Menzies et al. 2006). The donor for both *Bt9* and *Bt10* is PI 178383, a landrace collected in Turkey (Harlan 1950) and crossed to ‘Elgin’ to develop the respective lines for the differential set (Goates 1996). *Bt9* was shown to be distinct from *Bt10* by Steffan et al. (2017) and mapped to the long arm of chromosome 6D. Its position was refined by Wang et al. (2019) to a region between 456 and 471 Mbp. Muellner et al. (2020) mapped *Bt12* originating from PI 911333, a Turkish landrace (Goates 2004), to the short arm of chromosome 7D.

In order to avoid frequent breakdowns of resistances in cultivars, it is necessary to make new resistance sources available to breeders and broaden the genetic basis for bunt resistance. As *Bt11* has so far been overcome by only two races of the closely related dwarf bunt pathogen (*Tilletia controversa*), it is considered the most durable of the known bunt resistance genes (Goates 2012). A donor for *Bt11* is PI 166910 (Goates and Bockelman 2012; Goates 2012), a wheat accession collected in Tokat, Turkey, in 1948 (Harlan 1950). Despite its valuable characteristics in terms of bunt resistance (Goates and Bockelman 2012), *Bt11* has not been deployed in breeding yet (Goates 2012). We therefore developed segregating populations with wheat accessions PI 166910 and PI 554119 (M822123) as the resistance donors to map the resistance factor *Bt11* and thus contribute to the development of highly and durably resistant cultivars.

## Materials and methods

### Plant material

#### Mapping populations

We investigated four different bi-parental populations putatively segregating for bunt resistance gene *Bt11*. Two mapping populations (MPs) were developed through a reciprocal cross with PI 166910 as the resistant and ‘Rainer’ as the susceptible parent. Mapping populations MP-PR1 and MP-PR2 resulting from this reciprocal cross each consist of 120 $F_{5:7}$ recombinant inbred lines (RILs). The third mapping population, MP-PL, was composed of 160 $F_{5:7}$ RILs derived from the cross PI 166910 $\times$ ‘Lukullus’. The resistance donor for these first three populations, PI 166910, is an awned Turkish landrace with a winter growth habit collected in 1948 (Harlan 1950) and contains a unique resistance factor designated *Bt11* by Robert Metzger in 1986 (Goates and Bockelman 2012; Goates 2012). In addition, PI 166910 was postulated to harbour the *Bt7* and *Bt9* resistance alleles (Abdalla 1984). ‘Rainer’ and ‘Lukullus’ are Austrian winter wheat cultivars released by Saatzucht Donau GesmbH and CoKG in 2006 and 2008, respectively. They are adapted to Central European growing conditions and highly susceptible to common bunt.

The fourth mapping population MP-MM with 106 $F_{5:7}$ RILs was developed from a cross between the bunt differential line M822123 (wheat accession PI 554119, *Bt11*) and ‘Mulan’. M822123 was developed by Robert Metzger in Oregon, U.S.A from a cross between ‘Elgin’ and PI 166910. ‘Mulan’ is a bunt susceptible winter wheat cultivar released by Nordsaat Saatzucht GmbH in 2007 with adaptation to a broad range of environments ranging from the Scandinavian region to Eastern Europe.

Additionally, 16 wheat accessions (Online Resource 3) were sourced for genotyping and haplotype comparison only (see below).

#### Field experiments and disease scoring

RILs of MP-PR1, MP-PR2 and MP-PL were evaluated for CB resistance in 2019 and 2020, while population MP-MM was evaluated in 2015, 2016, 2020 and 2021. Field trials were artificially inoculated and sown as randomized complete block designs with two replications. Populations MP-PR1 and MP-PR2 were additionally evaluated in 2022 in a field trial sown as an augmented balanced incomplete block design with 23 and 33 out of 120 lines in each population, respectively, in two replications and the remaining 97 or 87 lines, respectively, in unreplicated plots. For this trial, a slightly different spore mixture was used compared to all other seasons of bunt evaluation. This bunt population was derived from infected spikes of the cultivar ‘Tilliko’ [released in 2016 by Cultivari gGmbH Darzau, AGES (2022)], a cultivar registered as bunt resistant. This bunt population is exceptionally virulent against resistance gene *Bt10* (Ritzer et al. 2022). Parental lines of the MPs, the set of bunt differential lines and the susceptible cultivar ‘Capo’ as a control were included in each of the field trials in order to monitor the virulence spectrum of the used inoculum. The experimental fields were located in Tulln, Austria (48${}^{\circ }$ 19′ 05″ N, 16${}^{\circ }$04′ 10″ E, elevation 177 m above sea level).

Field trials were sown in autumn, and all grain samples were inoculated prior to sowing with a teliospore mix representing the prevailing common bunt population in Austria in all years except 2022, were the bunt population described above was used. Inoculation was carried out following a protocol adapted from Muellner et al. (2020) and Goates (1996). Infected wheat ears were harvested from field plots with medium infection levels (20–50% incidence) and stored in a dry place at room temperature. A wide range of genotypes was used as a source for spore collection to avoid unintended selection. Spores were extracted from the ears, cleaned from plant residuals and mixed with a 0.2%-solution of methylcellulose in water. The resulting spore suspension was applied to grain samples in a concentration of 0.09 g of spores ($\equiv$ 0.3 ml of spore suspension) per 10 g of seeds. Field plots were sown with 10 g of seeds for each plot as double rows of 1.6 m in length and spaced 25 cm apart, resulting in plot sizes of approximately 1 $\hbox {m}^{2}$.

Heading date (HD) was scored when 50% of all spikes in a single plot had reached BBCH 55 and plant height (PH) was measured at maturity in  cm excluding awns. CB incidence (CBI) was recorded as the percentage of infected spikes out of 150 randomly chosen spikes per plot. Whether a spike was infected or not was determined by cutting it open and checking for bunt balls inside. Spikes were recorded as infected when at least one bunted spikelet was spotted.

#### Phenotypic analysis

Best linear unbiased estimates (BLUEs) were calculated for each genotype and trait in each individual experiment using a model of the form

1 $$\begin{aligned} P_{ik} = \mu + G_{i} + R_{k} + e_{ik} \end{aligned}$$

with $P_{ik}$ as the observed phenotypic value for the respective trait, $\mu$ as the grand mean, $G_{i}$ as the effect of the $i^{th}$ genotype, $R_{k}$ as the effect of the $k^{th}$ replication and $e_{ik}$ as the error term (BLUEs for each experiment are available in Online Resource 5). For analysis across environments, the model was extended to

2 $$\begin{aligned} P_{ijk} = \mu + G_{i} + E_{j} + E_{j}(R_{k}) + GE_{ij} + e_{ijk} \end{aligned}$$

taking also the effect of the $j^{th}$ year $E_{j}$, the nested effect of replication *k* in year *j* ($E_{j}(R_{k})$) and the genotype-environment-interaction $GE_{ij}$ into account. The grand mean and the genotype effect were treated as fixed effects, while all other effects were modelled as random in both models (1 and 2). For calculation of variance components, only the grand mean was modelled as a fixed effect. Models were fit with the remlf90 function in the R (R Core Team 2021) package breedR (Muñoz and Sanchez 2020).

Broad-sense heritability was calculated as suggested by Strube (1967) as

3 $$\begin{aligned} H^{2} = \frac{\sigma ^{2}_{\text G}}{\sigma ^{2}_{\text G} + \frac{\sigma ^{2}_{{\text {G}} \times {\text {E}}}}{n_{E}} +\frac{\sigma ^{2}_{e}}{n_{\text R} \cdot n_{\text E}} } \end{aligned}$$

with $\sigma ^{2}_{\text G}$ as the genotypic variance, $\sigma ^{2}_{{\text {G}} \times {\text {E}}}$ as the genotype-environment-interaction, $\sigma ^{2}_{e}$ as the residual variance, $n_{\text R}$ as the number of replications in each year and $n_{\text E}$ as the number of test environments. All statistical analyses were carried out in R (R Core Team 2021).

#### Genotypic data

Genome-wide SNP (single-nucleotide polymorphism) marker data was obtained for all parental lines and RILs in the MPs (raw data available in Online Resource 9). Additionally, 16 wheat accessions were genotyped with the purpose of confirming the pedigree of resistant lines and comparing haplotypes for QTL regions between carriers of *Bt11*, *Bt9* and susceptible lines, respectively (raw data available in Online Resource 10). Fresh leaf samples were used to extract genomic DNA following a protocol adapted from Saghai-Maroof et al. (1984). Ten samples were collected from each genotype, dried and pooled for DNA extraction. Genotyping was performed by TraitGenetics GmbH (Gatersleben, Germany, https://traitgenetics.com) with the Illumina Infinium 25 K XT array (Gogna et al. 2022) yielding 24145 SNP markers. Quality control was performed on the marker data for RILs prior to the construction of linkage maps and QTL anaylsis. For each MP, markers with more than 20% missing calls and markers showing significant ($p \le 0.001$) segregation distortion were discarded. Genotypes with more than 20% missing marker data were excluded from the analysis and genotypes with more than 95% identical marker calls were combined. Co-located markers were generally excluded, but one randomly chosen SNP of each set of co-located markers was kept to ensure maximum mapping resolution.

#### Linkage map construction

The R package ASMap (Taylor and Butler 2017) was used to determine linkage groups (LGs). For map construction, the sum of recombination events between markers was minimized by keeping the default setting of the objective function in the call to mstmap. LGs were identified with a stringent *p*-value threshold (from $p < 1 \times e^{-7}$ to $p < 1 \times e^{-11}$ depending on the MP) for marker clustering. LGs were assigned to wheat chromosomes by BLASTing markers against the IWGSC RefSeq v2.1 (Zhu et al. 2021) and obtaining their chromosome and basepair positions. Reordering of markers within robust LGs was performed using a less stringent *p*-value threshold. Map distances were calculated with the Kosambi mapping function based on recombination frequencies between markers. After a preliminary QTL scan, LGs identified as harbouring bunt resistance loci in this first scan were refined. LGs belonging to the same chromosome were combined to determine the location of respective QTL based on the whole chromosome. The order of these LGs was determined based on physical marker positions obtained from the IWGSC RefSeq v2.1 (Zhu et al. 2021) for markers in the individual LGs. Gaps between LGs forming a chromosome were defined by re-estimating the linkage map after merging individual LGs and thereby calculating the total chromosome length in cM. Marker coverage on chromosome 6D was comparably low and large gaps were present between individual LGs in all genetic maps on this chromosome. While ordering markers based on their physical positions in the IWGSC RefSeq v2.1 generally worked well, the marker orders within the LGs on the distal end of chromosome 6D harbouring a QTL were not in full agreement with marker orders based on physical positions in the reference sequence. To evaluate reliability of physical positions, we aligned markers in this specific 6DL region against other published chromosome assembled wheat genomes from the 10+ Wheat Genomes Project (https://10wheatgenomes.org) (Walkowiak et al. 2020). We blasted markers using BLASTn at https://galaxy-web.ipk-gatersleben.de and selected those hits on the reference sequences that had the highest query coverage and lowest expectation value per marker and genome. Genome sequences of CDC Stanley v1.2, Jagger v1.1, Julius v1.0, Mace v1.0, Norin61 v1.1 and SY Mattis v1.0 were used. Graphical representations of LGs were compiled using R package LinkageMapView (Ouellette et al. 2018).

#### QTL analysis

QTL analyses for common bunt resistance QTL were performed for each MP separately employing the population-specific linkage map. All analyses were conducted with the R package R/qtl (Broman et al. 2003). Analyses for individual years were conducted using BLUEs across replications while BLUEs across experiments were used for analyses across years. Main effect, heavy and light interaction penalties for LOD (logarithm of the odds) scores at different significance levels were determined by running 1000 permutations (Manichaikul et al. 2009) using Haley-Knott regression (Haley and Knott 1992). The multiple imputation method proposed by Sen and Churchill (2001) was applied to impute missing genotype information. To determine the optimal QTL model, the automated search algorithm implemented in the stepwiseqtl-function of the R/qtl-package was employed. The maximum number of QTL was set to seven. This procedure first applies forward selection, searching for additional additive or interacting QTL in each step until the maximum number of QTL is reached. Subsequently, backward elimination down to the null model is performed. Among all models, the one with the maximum penalized LOD score according to the penalties derived by permutation analysis is chosen as the optimal model. The penalized LOD criterion implemented in the stepwise-function requires strong evidence for additional QTL and as the detection of linked QTL is essential in to our study, we followed the suggestion in Broman and Sen (2011) and applied the most liberal penalties in the model search. Based on the results from the stepwise selection procedure, multiple QTL models (MQM) were fitted for individual experiments as well as for BLUEs across experiments. LOD scores, additive effect estimates and the amount of phenotypic variance explained by each QTL or interaction were derived from the drop-one-ANOVA table of the MQM analyses. As Broman and Sen (2011) recommend, Bayes’ credible interval was used to derive interval estimates for individual QTL. The nominal Bayes’ fraction was set to 95%.

#### Haplotype comparison

Physical positions and chromosome assignments for marker data of the 16 additional wheat accessions were obtained by BLASTing markers against the IWGSC RefSeq v2.1 as described in the section on linkage map construction. Accessions were sorted based on their *Bt*-gene postulations in order to compare allele calls between carriers of different resistance sources in specific chromosomal regions showing significant association with CBI in the QTL analyses. Gene postulations were either obtained from the GRIN database (available at https://npgsweb.ars-grin.gov/gringlobal/search), scientific publications or personal communications with breeders. The full list of accessions and references to *Bt*-gene postulations is available in Online Resource 3.

## Results

### Field trials

#### Common bunt race spectrum

**Table 1:** Virulence patterns of common bunt inocula on genotypes of the bunt differential set and the susceptible control ‘Capo’ across six years. Mean values of common bunt incidence (CBI) from two replications in each year (2015, 2016, 2019–2022)

Genotype	CBI 15	CBI 16	CBI 19	CBI 20	CBI 21	CBI 22$^{a}$
Capo (susceptible)	62.1	81.2	71.7	78.7	70	91.5
Sel2092 (*Bt1*)	0	1.9	0	0	0	0
Sel1102 (*Bt2*)	60.2	44.5	49	70.3	27.7	98
Ridit (*Bt3*)	1.6	2	5	0.7	4	15.5
CI1558B (*Bt4*)	3.7	2.2	5	2	2.7	4
Hohenheimer (*Bt5*)	0	0	0	1	0	10
Rio (*Bt6*)	1.8	0.2	2	0	5	4
Sel500-77 (*Bt7*)	71.5	51.5	52	50	26.7	98
M822161 (*Bt8*)	1	0.2	2.3	1.3	3.7	4
M90387 (*Bt9*)	0	0	4.3	0	10	5.5
M822102 (*Bt10*)	0	0.2	2	0	6.3	50
M822123 (*Bt11*)	0	0	0	0	0	0
PI199333 (*Bt12*)	0	0.5	0	8$^{b}$	0.3	0
Thule-III (*Bt13*)	6.8	2.5	28	7.3	12.3	30
PI173437 (*BtP*)	1.1	1	10	3.7	6	15.5

$^{a}$A different, more aggressive inoculum was used for inoculation in 2022

$^{b}$Contamination in the seed sample, score not reliable

To monitor behaviour of the bunt population across years, the spore mixture used for artificial inoculation of the MPs was tested in parallel on the bunt differential set in each year. Virulence patterns against the individual bunt resistance genes were comparable across years with some quantitative variation observed on the differential lines for *Bt2*, *Bt7*, *Bt13* and *BtP* (Table 1). Reactions of the differential lines for *Bt6*, *Bt9* and *Bt10* indicated slight changes in the virulence spectrum of the applied inoculum between 2020 and 2021. In 2022, a different and more aggressive spore mixture was applied to the seed samples, showing increased aggressiveness against *Bt3*, *Bt5* and *Bt10*. It has to be noted that field infection levels were generally higher in 2022 compared to all other years, presumably due to specific environmental conditions in this season (Online Resource 8). The differential line for *Bt11*, M822123, showed complete resistance in all plots. ‘Capo’, the cultivar used as the susceptible control, was highly infected in all years with CBI levels ranging between 62.1 and 91.5%.

**Table 2:** Means of parents, means and ranges for individual years and/or BLUEs across years, broad-sense heritability ($H^{2}$, across years) or repeatability (r, for individual experiments) and variance components for all analysed traits of all mapping populations (PR1 = PI 166910 $\times$ ‘Rainer’, PR2 = ‘Rainer’ $\times$ PI 166910, PL = PI 166910 $\times$ ‘Lukullus’, MM = M822123 $\times$ ‘Mulan’).

Experiment		Parents		Population					
		PI	Rai	Mean (range)	$H^{2}$(r)	$V_{\text G}$	$V_{\text {Env}}$	$V_{\text {GE}}$	$V_{\text e}$
PR1 (*n* = 120)									
CBI$^{b}$	2019	0	67.6	9.0 (0–74.7)	0.99$^{a}$	262			14.9
	2020	0	73.5	10.6 (0–90.0)	0.97$^{a}$	366			47.3
	2022	0	99	24.6 (0–99)	0.84$^{a}$	519			198
	Mean	0	79.1	12.9 (0–79.1)	0.97	383	29.6	32.5	49.3
PH$^{c}$	Mean	115	93	112 (91–127)	0.77	29	83.2	16.9	34.6
HD$^{d}$	Mean	29.3	26.5	28.2 (25.5–31.7)	0.92	1.5	29.6	0.2	0.7
PR2 (*n* = 120)									
CBI	2019	0	67.6	9.2 (0–74.7)	0.98$^{a}$	305			24
	2020	0	73.5	11.5 (0–98)	0.97$^{a}$	443			56
	2022	0	99	21.9 (0–99)	0.92$^{a}$	594			107
	Mean	0	79.1	12.2 (0–79)	0.97	424	17	24.5	49.7
PH$^{c}$	Mean	116	93	109 (93–126)	0.85	30.8	72	7.9	34.6
HD$^{d}$	Mean	29.3	26.5	28.2 (25.8–32)	0.89	1.1	30.3	0.3	0.6
PL (*n* = 160)									
CBI	2019	0	43.3	7.1 (0-72)	0.96$^{a}$	204			15.2
	2020	0	65.8	9.4 (0−88.7)	0.98$^{a}$	359			23.8
	Mean	0	55.5	8.1 (0−74.2)	0.95	261.6	1.8	20.4	19.4
PH	Mean	117	88	110 (88-128)	0.79	29.7	149.9	6.8	34.5
HD	Mean	30.1	29.1	29.8 (22.5-35)	0.91	3.8	39.8	0.5	0.8
MM (*n* = 106)									
CB	2015	0	40.5	7.9 (0−85.5)	0.99$^{a}$	297			2.7
	2016	0	28.1	6.6 (0−73.5)	0.99$^{a}$	239			15
	2020	0	25.3	6.6 (0−91.3)	0.97$^{a}$	213			31.2
	2021	0	13.7	7.1 (0−69.3)	0.94$^{a}$	165			22.5
	Mean	0	26.8	7.2 (0−70.5)	0.96	197.5	0.3	27.4	18.2
PH	Mean	95	82	96 (71-127)	0.97	78.3	58.6	8.6	16.4
HD	Mean	32.7	32	31.7 (27.7−37.3)	0.93	2.3	33.6	0.5	1.6

The number of RILs in each population is given in brackets. $V_{\text g}$ is the genotypic variance component, $V_{\text {env}}$ denotes the variance component explained by the year effect, $V_{\text {GE}}$ is the variance component for genotype-environment interaction and $V_{\text e}$ denotes the residual variance component

In 2022 (CB22), a spore mixture with different virulence was used for artificial inoculation

$$^{a}$$repeatability

$^{b}$ common bunt incidence in %

$^{c}$ plant height in cm

$^{d}$ heading date in days after April 30

#### Phenotypic traits

**Fig. 1:**
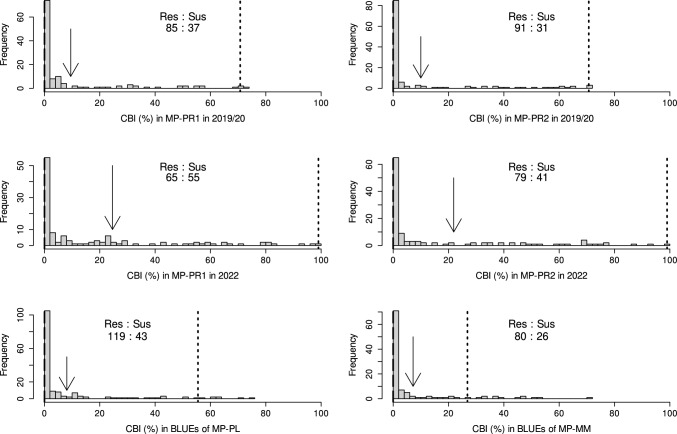
Histograms for all mapping populations (MP) with ratios indicating the proportion of resistant (Res, < 5% infection) to susceptible (Sus, $\ge$ 5% infection) RILs per population. MP-PR1 and MP-PR2 were inoculated with a different bunt population in 2022 which was more aggressive, and therefore, histograms are shown for BLUEs across 2019 and 2020 (row 1) and for 2022 (row 2) separately for these two MPs. For MP-PL and MP-MM, histograms for best linear unbiased estimators (BLUEs) across years are shown (row 3). Arrows indicate the population mean in the respective data set, dashed lines indicate CB levels of the resistant and dotted lines of the susceptible parent

CBI was highly heritable across years with estimates of $H^{2}=0.95$ or $H^{2}=0.97$ for individual MPs (Table 2). Estimates of $H^{2}$ for PH and HD showed more variability compared to CBI and ranged between 0.77 and 0.97. Average CBI levels were similar across populations and experiments except for trials conducted in 2022 which showed levels of CBI approximately 50% higher compared to previous years. High variation in phenotypic values was observed for all traits and MPs with CBI ranging between 0  and 99%, PH ranging from 65  to 145 cm and HD varying between May 21 and June 13. The resistant parental lines of the MPs (PI 166910 and M822123) were taller and had a slightly later HD than the susceptible parents (‘Rainer’, ‘Lukullus’ or ‘Mulan’). MPs with PI 166910 as the resistance donor had similar average PH and HD, while RILs in MP-MM with M822123 as the donor line were on average shorter and had later HD. Both resistant parents showed no bunt infections at all, while especially ‘Rainer’ was highly infected (67.6−99%), followed by ‘Lukullus’ (43.3–65.8%) and ‘Mulan’, the latter being moderately infected (13.7–40.5%, Fig. 1). ANOVA revealed that the genotypic effect explained most of the variance observed for CBI, while variance components for environmental effects, genotype-environment interaction and error variance were small. Variation in PH and HD, on the other hand, was for the largest part explained by the environmental effect. The only exception from this pattern was PH in MP-MM, for which the genotypic variance was larger than the environmental variance. CBI was negatively correlated with PH in all MPs with correlation coefficients ranging from $r=-0.13$ in MP-MM to $r=-0.25$ in MP-PR1 across years (Table 3). No correlation was observed for CBI with HD except in MP-PR1 where a low but significant negative correlation of $r=-0.12$ was found. HD and PH were positively correlated (coefficients ranging from $r=0.55$ in MP-PR2 to $r=0.71$ in MP-PL) in all MPs except for MP-MM in which the two traits were negatively correlated ($r=-0.18$). The parental lines for RILs in MP-MM had approximately the same HD and were also more similar in PH compared to parents in all other MPs.

**Fig. 2:**
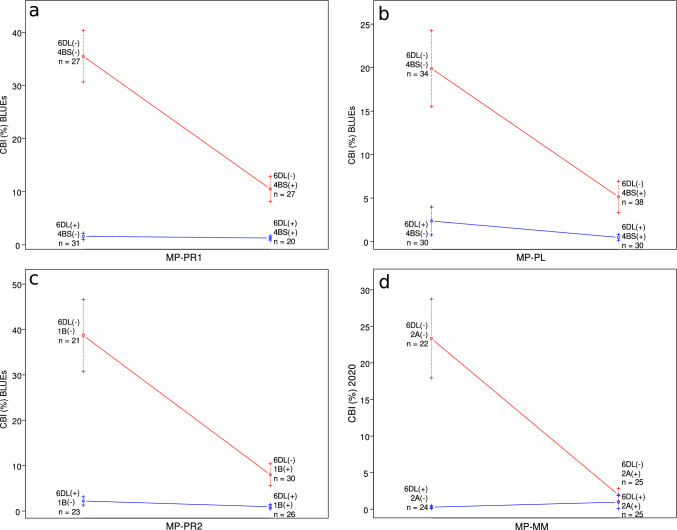
Epistatic interactions between the two QTL with the largest effects in the individual mapping populations (MPs). **a** and **b** in the first row show epistatic interactions between *QBt.ifa-6DL* and *QBt.ifa-4BS* in MP-PR1 (**a**) and MP-PL (**b**). Interactions between *QBt.ifa-6DL* and *QBt.ifa-1BS* in MP-PR2 (**c**) and *QBt.ifa-2A* in MP-MM (**d**) are shown in the second row. All effectplots show data for BLUEs across experiments, except **d** which shows data from 2020 as *QBt.ifa-2A* was not detected in BLUEs across years in MP-MM. Standard errors are indicated by error bars and numbers next to the bars designate the number of lines harbouring the respective QTL(-combination)

**Fig. 3:**
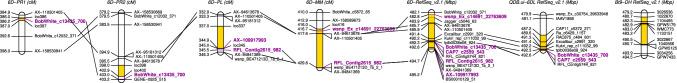
Comparison of linkage maps for the distal end of chromosome 6D from our mapping populations (6D-PR1, 6D-PR2, 6D-PL and 6D-MM) with physical positions (Mbp) of the markers on the IWGSC RefSeq v2.1 (Zhu et al. 2021) (6D-RefSeq_v2.1), the physical positions (RefSeq v2.1) of markers in the linkage map for the 6D QTL identified by Wang et al. (2019) (QDB.ui-6DL RefSeq_v2.1) and markers to the 6D QTL published by Steffan et al. (2017) (Bt9-DH RefSeq_v2.1). Markers highlighted in magenta indicate peak markers in individual MPs: *BobWhite_c13435_700* in MP-PR1 and MP-PR2; *AX-109917993* in MP-PL; *wsnp_Ex_c14691_22763609* and *RFL_Contig2615_982* in MP-MM; *CAP7_c2559_543* for *QDB.ui-6DL*. Regions marked in yellow indicate the 6D QTL-region across experiments in the individual MP or study

**Table 3:** Pearson’s correlation coefficients between heading date in days after April 30 (HD), plant height in centimeters (PH) and common bunt incidence levels in percent (CBI) of individual mapping populations

MP-PR1: ‘Rainer’ $\times$ PI 166910 (*n* = 122)			MP-PR2: PI 166910 $\times$ ‘Rainer’ (*n* = 122)		
	PH	CBI		PH	CBI
HD	0.62	− 0.12	HD	0.55	n.s.
CBI	− 0.25		CBI	− 0.19	

MP-PL: PI 166910 $\times$ ‘Lukullus’ (*n* = 162)			MP-MM: M822123 $\times$ ‘Mulan’ (*n* = 106)		
	PH	CBI		PH	CBI
HD	0.71	n.s.	HD	− 0.18	n.s.
CBI	− 0.19		CBI	− 0.13	

The number of lines in each population is given in brackets. Correlation coefficients were calculated based on best linear unbiased estimators across years. Non-significant correlations are indicated by n.s., all other correlation coefficients were significant at the $p < 0.001$ level

### QTL analysis

#### Linkage maps

During the data preparation for linkage map construction, we noticed that the set of markers being polymorphic between the parental genotypes differed by more than 1000 markers when comparing MP-PR1 and MP-PR2. We therefore conclude that the two crosses between ‘Rainer’ and PI 166910 were actually not reciprocal, but that the individuals of PI 166910 used as mother/father in the crosses rather have to be treated as two sublines. All subsequent analyses were therefore performed separately in the two MPs instead of treating all RILs as one homogeneous MP. The linkage map for population MP-PR1 was built on data from 105 RILs with 2430 markers and comprised 32 LGs, resulting in a total map length of 3792.4 cM. In MP-PR2, 100 genotypes with 2795 markers were used to construct a linkage map comprising 44 LGs, summing up to a total length of 4574 cM. For population MP-PL, 132 RILs were available after quality control. The map for MP-PL consisted of 2114 markers in 38 LGs with a total map length of 4230.1 cM. In population MP-MM, 96 RILs and 1821 markers passed quality control. The linkage map consisted of 31 LGs and was 3160.1 cM long. All wheat chromosomes were represented by the LGs in each of the four individual maps, which are available in Online Resource 1.

#### QTL identification and analysis

Wheat chromosome 6D was identified as harbouring a locus controlling resistance against common bunt in all four MPs (Table 4, Fig. 3, Online Resource 6). The QTL mapped to the long arm of chromosome 6D and is subsequently referred to as *QBt.ifa-6DL*. It was detected consistently in all experiments and MPs, had a major effect on CBI and explained between 18.5% (MP-PL, 2020) and 49.6% (MP-PR1, 2022) of the phenotypic variation with an average of 33.9%. The QTL spanned a maximum genetic distance of 13.9 cM (482.8–495.2 Mbp) across MPs, flanked by markers *wsnp_Ex_c14691_22763609* and *AX-94841369*. Comparison of the *QBt.ifa-6DL* region with six additional reference sequences of the 10+ Wheat Genomes Project suggested that the QTL interval might be smaller than the region based on IWGSC RefSeq v2.1 (Zhu et al. 2021). Maximum physical distance between peak markers in individual MPs was approximately 2.580 Mbp in RefSeq v2.1, while distances between these markers ranged between 0.865 Mbp (CDC Stanley) and 2.369 Mbp (Julius) in the additional reference sequences (Online Resource 4).

On chromosome 4B, two regions were associated with common bunt resistance. The first locus, hereafter referred to as *QBt.ifa-4BS*, is located on the short arm of chromosome 4B and was found in all data sets for MP-PR1 and MP-PL as well as in MP-MM in 2015. The second locus will be referred to as *QBt.ifa-4BL* and mapped to the long arm of chromosome 4B. It was identified in MP-MM in 2016. In MP-PR2, a locus with a peak marker on the short arm of chromosome 4B was associated with CBI, but the estimate derived using Bayes’ credible interval for this locus spanned almost the whole LG, so no unambiguous assignment to either *QBt.ifa-4BS* or *QBt.ifa-4BL* was possible. However, if the interval estimate was based on LOD drop-off, the region was corresponding to *QBt.ifa-4BS*. The effect of *QBt.ifa-4BS* on CBI was on average smaller compared to *QBt.ifa-6DL*. *QBt.ifa-4BS* explained between 16.5% (MP-PR1, 2022) and 28.3% (MP-MM, 2015) of the total phenotypic variance, averaging 22.0% across experiments. In MP-PL, *QBt.ifa-4BS* explained a larger part of the total phenotypic variance than *QBt.ifa-6DL* in two out of three data sets, while *QBt.ifa-6DL* explained higher amounts of the total variation in all other MPs and experiments. *QBt.ifa-4BS* mapped to a region between 11.5 and 28.6 Mbp across MPs, spanning a maximum distance of 38 cM on the short arm of chromosome 4B flanked by markers *wsnp_BF483640B_Ta_2_2* and *AX-94707905*. Resistance improving alleles for these loci on chromosomes 4B and 6D descended from M822123 (MP-MM) or PI 166910 (all other MPs), respectively.

Apart from *QBt.ifa-6DL* and *QBt.ifa-4BS* that were associated with CBI in three or all four MPs, additional loci were detected in single MPs. QTL on chromosomes 1A (*QBt.ifa-1A*), 1B (*QBt.ifa-1B*) and 7B (*QBt.ifa-7B*, on LG 7.2) were identified in MP-PR2. *QBt.ifa-1B* was detected in all years and BLUEs across years and had the second largest effect on CBI levels in MP-PR2, while the loci on chromosomes 1A and 7B were significant in two out of four experiments. Both *QBt.ifa-1A* and *QBt.ifa-7B* had minor effects on CBI with an average of 6.1% (*QBt.ifa-1A*) and 14.2% (*QBt.ifa-7B*) of the total variance explained by the QTL. *QBt.ifa-1B* had a larger effect and explained 31.1% (2019) to 42.3% (2020) of the total phenotypic variance in MP-PR2. The interval estimate for *QBt.ifa-1B* varied between data sets, with the genetic distance spanned by the QTL ranging between 7.6-18 cM in 2019 and 31-34 cM in 2020 and corresponding to a region between 12.2 and 46.9 Mbp on the short arm of chromosome 1B. This region was flanked by markers *BS00004903_51* and *Tdurum_contig9762_314*. An additional QTL on chromosome 2A (*QBt.ifa-2A*) was identified in MP-MM. It was detected in two out of five experiments and was located on the proximal end of the short arm of chromosome 2A , flanked by markers *Tdurum_contig29983_490* and *AX-94381641*. *QBt.ifa-2A* had the second largest effect in MP-MM in 2020 and explained 26.5% of the total variation across data sets. *QBt.ifa-4BL* was only detected in MP-MM in 2016 and explained 10.2% of the phenotypic variance. The QTL spanned a distance of 14 cM on chromosome 4BL, corresponding to a region between 662.9 Mbp and 671.4 Mbp. Epistatic interactions were found for *QBt.ifa-6DL* with all other bunt QTL except for *QBt.ifa-1A* and *QBt.ifa-4BL* as well as between *QBt.ifa-1B* and *QBt.ifa-7B* (Fig. 2).

Flanking markers for all identified QTL in all mapping populations are provided in Online Resource 2.

### Haplotype comparisons

A comparison of SNP haplotypes of the chromosomal region at and surrounding *QBt.ifa-6DL* was performed. For this purpose, the SNP allele calls of 21 wheat genotypes comprising the five parents of the MPs and 16 additional wheat accessions were compared side by side (Online Resource 3). Four genotypes postulated to possess the *Bt11* allele, two genotypes with *Bt8*, three genotypes with *Bt9*, the *Bt10* differential line as well as five genotypes with different combinations of *Bt8*, *Bt9* and *Bt10* were included. In addition, we also compared haplotypes of six susceptible cultivars. Differences in haplotypes in the 6DL region were observed between *Bt9* genotypes and accessions harbouring *Bt11*, while no clear pattern was found for *Bt8*- and *Bt10*-lines. The SNP haplotype of PI 166910 in the *QBt.ifa-6DL* region was found in all the genotypes postulated to harbour *Bt11*, including the *Bt11* differential line.

**Table 4:** Effect estimates and chromosomal locations in cM and Mbp as well as peak markers for common bunt QTL identified in individual mapping populations (MPs) using a forward-backward iteration for multiple QTL mapping

Experiment	Chrom.	Interval estimate		Peak position					Peak marker
		cM	Mbp$^{c}$	cM	Mbp	$\mathrm {Add^{a}}$	PV (%)^b^	LOD	
MP-PR1$^{d}$ (*n* = 105)									
2019	4B	8.7–23	13.6–26.2	13.0	13.6	5.6	23.1	8.2	Excalibur_c64418_447
2020	— $_{\text {''}}$ —	8.7–23	13.6–26.2	1 6.0	13.6	6.8	24.1	8.5	— $_{\text {''}}$ —
2022	— $_{\text {''}}$ —	6–20	11.5–26.2	8.0	13.6	7.8	16.5	7.1	— $_{\text {''}}$ —
BLUEs	— $_{\text {''}}$ —	8–22	13.0–26.2	8.7	13.6	6.4	21.3	8.5	— $_{\text {''}}$ —
2019	6D	386–387	492.6	386.2	492.6	8.4	39.1	12.5	BobWhite_c13435_700
2020	— $_{\text {''}}$ —	386–387	492.6	386.2	492.6	9.6	38.6	12.4	— $_{\text {''}}$ —
2022	— $_{\text {''}}$ —	386–387	492.6	386.2	492.6	16.0	49.6	16.8	— $_{\text {''}}$ —
BLUEs	— $_{\text {''}}$ —	386–387	492.6	386.2	492.6	11.0	45.7	15.4	— $_{\text {''}}$ —
Epistatic Interactions				Add	PV%	LOD			
2019	4B:6D			5.5	10.8	4.2	46.5	14.3	Simultaneous fit
2020	— $_{\text {''}}$ —			6.7	10.6	4.1	46.5	14.2	— $_{\text {''}}$ —
2022	— $_{\text {''}}$ —			7.5	7.7	3.6	54.5	17.9	— $_{\text {''}}$ —
BLUEs	— $_{\text {''}}$ —			6.4	10.2	4.4	52.6	17.0	— $_{\text {''}}$ —
MP-PR2$^{d}$ (*n* = 100)	Chr	cM	Mbp	cM	Mbp	Add	PV%	LOD	Marker
2022	1A	65–162	549.1–94.8	126.1	500.3	5.7	5.4	4.0	wsnp_Ex_rep_c81556_76277906
BLUEs	— $_{\text {''}}$ —	116.8–165.4	515.2–355.2	158.0	403.9	5.2	6.7	4.2	AX-94404955
2019	1B	7.6–18	12.2–41.5	11.8	28.2	7.0	31.1	12.0	AX-158570920
2020	— $_{\text {''}}$ —	31–34	44.6–46.9	31	46.1	7.2	42.3	19.4	Tdurum_contig55639_241
2022	— $_{\text {''}}$ —	11.8–16	28.2–43.2	11.8	28.2	10.6	32.0	16.9	AX-158570920
BLUEs	— $_{\text {''}}$ —	14–18	28.2–43.2	11.8	28.2	8.6	33.9	15.9	— $_{\text {''}}$ —
2022	4B	3.8–184	9.2–664.0	28.0	19.0	4.7	3.6	2.7	tplb0035d20_506
BLUEs	— $_{\text {''}}$ —	18–205.8	15.0–671.0	28.0	19.0	4.4	5.0	3.2	— $_{\text {''}}$ —
2019	6D	398–400	485.7–492.6	403.0	492.6	9.0	39.3	14.3	BobWhite_c13435_700
2020	— $_{\text {''}}$ —	398–402	485.7–492.6	403.0	492.6	8.6	36.9	17.7	— $_{\text {''}}$ —
2022	— $_{\text {''}}$ —	400–402	485.7–492.6	403.0	492.6	16.0	49.1	22.4	— $_{\text {''}}$ —
BLUEs	— $_{\text {''}}$ —	398–400	485.7–492.6	403.0	492.6	11.4	44.3	19.1	— $_{\text {''}}$ —
2019	7B	108–132.9	26.4–7.1	116.5	12.8	4.4	6.8	3.3	AX-158593396
2020	— $_{\text {''}}$ —	118–122	12.8–10.1	121.7	10.1	4.3	21.6	12.0	AX-94810990
Epistatic Interactions				Add	PV%	LOD			
2019	1B:6D			8.3	16.8	7.3	57.9	18.8	Simultaneous fit
2020	— $_{\text {''}}$ —			6.6	9.6	6.1	70.6	26.6	— $_{\text {''}}$ —
2020	1B:7B			7.5	13.0	8.0			
2020	7B:6D			5.6	6.3	4.2			
2022	1B:6D			10.6	14.2	9.1	72.8	28.3	Simultaneous fit
BLUEs	— $_{\text {''}}$ —			9.8	17.7	9.7	68.7	25.2	— $_{\text {''}}$ —
MP-PL$^{d}$ (*n* = 132)	Chr	cM	Mbp	cM	Mbp	Add	PV%	LOD	marker
2019	4B	10–45	15.8–28.6	14.9	15.8	4.6	21.5	8.1	AX-94629926
2020	— $_{\text {''}}$ —	7–15	13.1–18.9	14.9	15.8	6.3	20.2	7.3	— $_{\text {''}}$ —
BLUEs	— $_{\text {''}}$ —	7–40	13.1–28.6	14.9	15.8	5.5	21.2	7.8	— $_{\text {''}}$ —
2019	6D	338–345	486.1–494.7	344.9	495.2	6.0	23.0	8.6	AX-109917993
2020	— $_{\text {''}}$ —	337–344.9	486.1–495.2	344.9	495.2	7.2	18.5	6.8	— $_{\text {''}}$ —
BLUEs	— $_{\text {''}}$ —	338–345	486.1–494.7	344.9	495.2	6.6	20.8	7.7	— $_{\text {''}}$ —
Epistatic Interactions				Add	PV%	LOD			
2019	4B:6D			5.0	9.2	3.7	34.4	12.1	Simultaneous fit
2020	— $_{\text {''}}$ —			6.0	7.5	2.9	30.3	10.3	— $_{\text {''}}$ —
BLUEs	— $_{\text {''}}$ —			5.5	8.4	3.4	32.6	11.3	— $_{\text {''}}$ —
MP-MM$^{d}$ (*n* = 96)	Chr	cM	Mbp	cM	Mbp	Add	PV%	LOD	Marker
2016	2A	0–4	0.3–35.1	0.4	31.8	5.0	28.3	10.5	AX-94684111
2020	— $_{\text {''}}$ —	0–5	0.3–35.1	0.4	31.8	4.8	24.7	7.1	— $_{\text {''}}$ —
2015	4B	24–34	19.3–27.3	24.6	17.8	7.9	28.3	8.4	tplb0060n12_565
2016	— $_{\text {''}}$ —	116–130	662.9–671.4	124.0	671.4	5.9	10.2	4.4	AX-158542232
2015	6D	416–428	482.8–494.7	416.7	482.8	6.0	29.7	8.8	wsnp_Ex_c14691_22763609
2016	— $_{\text {''}}$ —	423–428	486–494.7	429.9	494.7	8.8	39.3	13.5	RFL_Contig2615_982
2020	— $_{\text {''}}$ —	424–429.9	486.1–494.7	429.9	494.7	6.9	29.7	8.3	— $_{\text {''}}$ —
2021	— $_{\text {''}}$ —	421–429.9	486–494.7	429.9	494.7	6.4	19.1	4.4	— $_{\text {''}}$ —
BLUEs	— $_{\text {''}}$ —	420–429.9	486–494.7	429.9	494.7	7.3	19.7	4.6	— $_{\text {''}}$ —
Epistatic Interactions				Add	PV%	LOD			
2015	4B:6D			7.5	13.4	4.4	43.1	11.7	Simultaneous fit
2016	2A:6D			7.7	18.1	7.3	56.7	17.5	— $_{\text {''}}$ —
2020	2A:6D			6.4	14.5	4.5	39.3	10.4	— $_{\text {''}}$ —
2021							19.1	4.4	— $_{\text {''}}$ —
BLUEs							19.7	4.6	— $_{\text {''}}$ —

The number of lines in each MP is given in brackets. Rows showing ‘simultaneous fit’ in the last column indicate the amount of phenotypic variance (column 8) and the LOD score (column 9) for the respective experiment (column 1) if all QTL significant in the data set were fit together

In 2022, a spore mixture with different virulence was used for artificial inoculation

$^{a}$Positive additive effects indicate a decreasing effect of the resistance-conferring allele

$^{b}$Percentage of phenotypic variance explained by the respective QTL

$^{c}$markers closest to the cM locations of the estimated borders for each QTL interval were used to determine interval regions in Mbp

$^{d}$PR1 = PI 166910 $\times$ ‘Rainer’; PR2 = ‘Rainer’ $\times$ PI 166910; PL = PI 166910 $\times$ ‘Lukullus’; MM = M822123 $\times$ ‘Mulan’

## Discussion

With a constant increase in organic or low-input farming systems and reports of resistance break-downs of common bunt resistant wheat cultivars, more and more stakeholders recognize that bunt pathogens should be considered in their agenda. In order to achieve sustainable bunt management that is successful in the long term, we regard the diversification of resistance sources available for applied breeding as essential. In this study, we therefore aimed at unlocking resistance loci originating from the Turkish landrace PI 166910 which was used to develop the differential line for bunt resistance gene *Bt11*. PI 166910 is postulated to carry three different *Bt*-genes: *Bt7*, *Bt9* and *Bt11* (Abdalla 1984). Goates (2012) called *Bt11* “the most difficult bunt resistance gene to overcome”. PI 166910 therefore constitutes an ideal source for additions to the range of bunt resistance sources for wheat breeding.

High infection levels in the susceptible check cultivar ‘Capo’ in all experiments showed that artificial infection was successful and disease pressure in all trials was high (Table 1). Unusually high infection levels in the susceptible control as well as in some of the bunt differential lines in 2022 can be explained by two main factors: (1) weather conditions in the critical time period for field infections were ideal in autumn 2021 with dry and cool soil into which seed samples were sown (Borgen 2000; Johnsson 1992) (Online Resource 8); (2) a slightly different spore mixture was used for artificial inoculation of seed samples for 2022 field trials. The primary source for this spore mixture were infected spikes of the cultivar ‘Tilliko’ which was bred to be bunt tolerant (AGES 2022; RWA 2023). As bunt populations with the ability to overcome previously effective host plant resistance factors are of special interest for research projects, this inoculum source was multiplied and used as an infection source for field trials. Although it is not known what kind of genetic source confers the tolerance of ‘Tilliko’, a comparison with the standard bunt population used before 2021 shows that the inoculum collected from ‘Tilliko’ is especially virulent against the differential lines for *Bt2* and *Bt10* (Table 1).

Repeatabilities for individual experiments were high, as well as heritabilities across years for individual MPs (Table 2). The largest part of the total observed variation in CBI levels was due to the genetic component, while year effects were the main source of variation for PH and HD. Similar results were obtained by other mapping studies investigating bunt diseases in wheat, e.g. Muellner et al. (2020, 2021); Steffan et al. (2017) or Wang et al. (2019). Distributions of CBI levels were strongly right-skewed in all MPs with around three quarters of all RILs per MP showing less than 5% CBI (Fig. 1). Such distributions are indicative of two resistance factors—in our MPs these were *QBt.ifa-6DL* and *QBt.ifa-4BS*, *QBt.ifa-1B* or *QBt.ifa-2A*, respectively, acting together (Fig. 2, Online Resource 7).

### QTL for common bunt resistance

#### QBt.ifa-6DL

Steffan et al. (2017) mapped bunt resistance gene *Bt9* to the distal end of wheat chromosome 6D using DArT markers (SNPs and presence-absence variants) in a doubled haploid (DH) population resulting from the cross PI 554099 (*Bt9*-differential) $\times$ ‘Cortez’. This *Bt9*-locus explained between 37.7 and 53.7% of the phenotypic variation for CBI in their trials. Wang et al. (2019) identified a QTL conferring dwarf bunt resistance in a DH population derived from a cross between IDO835 (a resistant breeding line) and ‘Moreland’ (susceptible cultivar (Souza et al. 2004)). The QTL was located on chromosome 6DL and explained 17–53% of the phenotypic variation in dwarf bunt incidence in their study. Resistance in these DH lines originated from UT944157, a sib-selection to the highly resistant cultivar ‘Golden Spike’ (Chen et al. 2018). We obtained physical positions according to IWGSC RefSeq v2.1 (Zhu et al. 2021) for markers to the 6DL loci published by Steffan et al. (2017) and Wang et al. (2019) via GrainGenes BLAST (Yao et al. 2022) (available at https://wheat.pw.usda.gov/). For markers to *QDB.ui-6DL* identified by Wang et al. (2019), FASTA sequences were obtained from JBrowse at the Wheat@URGI portal (Alaux et al. 2018) (available at https://urgi.versailles.inra.fr/jbrowseiwgsc/gmod_jbrowse/?data=myData/IWGSC_RefSeq_v1.0) before blasting them on GrainGenes. These most recent physical positions for the two previously published 6DL loci were compared to the QTL region of *QBt.ifa-6DL* and visualized in Figure 3. Both Wang et al. (2019) and Steffan et al. (2017) suggest that the QTL they found on 6DL corresponds to *Bt9*. *QBt.ifa-6DL* detected in our experiments is located very close to or partially overlapping with these two previously identified 6D loci (Fig. 3, Online Resource 3). The exact location cannot be determined in our MPs as polymorphic markers at the distal end of chromosome 6D are scarce. In MP-PR1 and MP-PR2, the most distal marker according to physical positions in RefSeq v2.1 was *BobWhite_c13435_700*. Figure 3 shows other markers mapping to positions beyond *BobWhite_c13435_700* in the linkage maps for MP-PR1 and MP-PR2, but these are possibly incorrectly ordered in the linkage group when compared to physical positions. As no polymporhic SNPs are available beyond the 492.6 Mbp-position, it cannot be determined whether the LOD-peak would appear at a more distal locus in MP-PR1 and MP-PR2 if more polymorphisms were available, or if *BobWhite_c13435_700* would still remain the peak marker in that case. Such a shift of the LOD peak to a more distal position in MP-PR1 and MP-PR2 seems plausible because of the peak marker locations in MP-PL (495.2 Mbp) and MP-MM (494.7 Mbp). Nevertheless, *BobWhite_c13435_700* is neither polymorphic in MP-PL and MP-MM, nor are polymorphic markers at very similar physical positions available in the linkage maps. In consequence, we hypothesize that LOD peaks would appear in more similar positions in the four MPs with a higher density of polymorphic SNPs at the distal end of 6DL. This marker scarcity at the distal chromosome end of 6D is a common problem, as Wang et al. (2019) similarly found no polymorphic markers in their MP beyond 492.6 Mbp on chromosome 6D (Jianli Chen and Pabitra Joshi, personal communication). Interestingly, Wang et al. (2019) described that their resistance donor is a sib-selection to the bunt resistant cultivar ‘Golden Spike’ (Hole et al. 2002a) and has the resistance conferring allele for their 6DL-2 marker (*Cap7_c2559_543*). They state that ‘Golden Spike’ has the *Bt9* resistance according to B.J. Goates, but no empirical evidence for this gene postulation is available to our knowledge. Nevertheless, it seems plausible based on the ‘Golden Spike’ pedigree and is supported by a matching haplotype of ‘Golden Spike’ with the haplotypes of the *Bt9*-differential, PI  554099 and Golden Spike’s resistance donor, PI 178383, in the *QDB.ui-6DL* region (469.83 Mbp - 471.02 Mbp based on IWGSC RefSeq v1.0) mapped by Wang et al. (2019) (Online Resource 3). PI  166910, the resistance donor for both our MPs in which *Cap7_c2559_543* and *BobWhite_c13435_700* are polymorphic and segregating, has the contrasting alleles to ‘Golden Spike’ (Online Resource 3) for these markers. This allele contrast can be interpreted as an indication that, as hypothesized above, the true peak location for *QBt.ifa-6DL* could actually be located in a more distal position in MP-PR1 and MP-PR2 and *BobWhite_c13435_700* was only identified as peak marker because it was the most distal SNP available in these MPs. In conclusion, PI 166910 most likely does not harbour *Bt9* since it has the susceptible alleles for the *Bt9*-markers identified by Wang et al. (2019). The causal gene for its resistance could be located at the more distal position on 6DL indicated by MP-PL and MP-MM. When comparing haplotypes in the 6DL region, it is striking that all genotypes postulated to harbour *Bt11* show a distinct allele pattern for markers between 494.58 Mbp and 494.69 Mbp which is different from all genotypes indicated to have the *Bt9* allele (Online Resource 3). Based on SNP markers on the 25K array, no universal functional markers for *QBt.ifa-6DL* were found. Therefore, for breeding purposes in new populations, parental testing and choosing appropriate selection markers is necessary. We recommend searching for markers in the potential *Bt11* region on the distal end of chromosome 6DL flanked by markers *BS00070856_51* and *AX-94841369*. According to the haplotypes of the resistance donors of our four MPs, PI 166910 and M822123 (PI 554119), we propose *Bt11* as the most likely causal gene underlying *QBt.ifa-6DL*. The fact that *QBt.ifa-6DL* is partially overlapping with the loci identified by Wang et al. (2019) and Steffan et al. (2017) does not necessarily contradict this hypothesis but could be explained by the coarse mapping resolution on 6DL resulting from marker scarcity. Our data suggest that *Bt9* and *Bt11* comprise either two genes in close neighborhood or two different alleles of the same locus on the distal end of chromosome 6DL. Except for accession PI 211657, all *Bt11*-lines listed in Online Resource 3 descend from PI 166910. Its genetic background can be expected to be present in all our RILs because M822123 is a cross between PI 166910 and ‘Elgin’. PI 166910 is also designated to harbour *Bt7*, but we can exclude this resistance type as the underlying factor for *QBt.ifa-6DL* since *Bt7* is not active against our inoculum (Table 1). Another source for bunt resistance that has been identified on wheat chromosome 6D is *Bt10*. Menzies et al. (2006) mapped *Bt10* to the short arm of 6D, which was confirmed in a study by Singh et al. (2015) investigating offspring from the cross ‘Carberry’ $\times$ ‘AC Cadillac’. This 6DS locus found in both studies is distinct from *QBt.ifa-6DL* and maps to a different location on the chromosome.

#### QBt.ifa-4BS

The QTL region for *QBt.ifa-4BS*, identified in all MPs, is partially overlapping with *QBt.ifa-4B* published by Muellner et al. (2020) (20.6–706.5 Mbp vs. 11.5–28.6 Mbp in our study). Similar to our results, Muellner et al. (2020) also had difficulties narrowing down the QTL region on the 4B chromosome as shown by the large physical interval. In their MPs, *QBt.ifa-4B* was detected in two out of five data sets (2015 and 2016) and explained 10.8 and 11.2% of the phenotypic variance. Interestingly, in MP-MM *QBt.ifa-4BS* was also only detected in data from 2015 and 2016 in our study. Across all MPs, it explained between 10.2 and 24.1% of the total variance in CBI. While *QBt.ifa-4B* was classified as a minor QTL by Muellner et al. (2020) which was not verified in their validation populations, it had a larger effect on CBI in all MPs except for MP-PR2 in our study. In MP-PL, the effect of *QBt.ifa-4BS* was even larger in some years than the effect of *QBt.ifa-6DL*, which was the main resistance source in all other MPs. We also observed epistatic interactions between *QBt.ifa-4BS* and *QBt.ifa-6DL* which was not the case in Muellner et al. (2020). Singh et al. (2015) also mapped a QTL conferring common bunt resistance to a region on the short arm of chromosome 4B which is overlapping with *QBt.ifa-4BS*, but they detected a significant effect of this locus in a single year only. The 4B locus explained 7.6% of the phenotypic variation in that year, its effect being thereby considerably smaller compared to *QBt.ifa-4BS*.

#### QBt.ifa-1B

In MP-PR2, which consists of RILs from a cross between ‘Rainer’ and a subline of PI 166910, we found a locus conferring resistance to common bunt on the short arm of chromosome 1B. This QTL had a strong effect on CBI in MP-PR2, explaining between 31.3 and 42.3% of the total phenotypic variation. In 2019, its effect was even stronger than the one of *QBt.ifa-6DL*. In 2022, RILs from MP-PR1 and MP-PR2 were tested with a different, more aggressive inoculum, which led to a higher number of susceptible RILs (Fig. 1). The increase in RILs with CBI levels above the 5%-threshold was lower in MP-PR2 compared to MP-PR1. Possibly, *QBt.ifa-1B*, due to its strong effect on CBI, together with *QBt.ifa-6DL* led to a more stable resistance in MP-PR2 RILs compared to lines in MP-PR1. The effect of *QBt.ifa-4BS* as an additional resistance source besides *QBt.ifa-6DL* in MP-PR1 RILs was weaker than the effect of *QBt.ifa-1B* which might be an explanation for the difference observed between the two MPs in 2022 (Online Resource 7). A locus conferring resistance to CBI in a similar region as *QBt.ifa-1B* was also found by Singh et al. (2015) in two out of three data sets included in their study. It explained 5 and 18% of the total phenotypic variation, respectively, and showed epistatic interactions with other bunt resistance loci detected on chromosomes 4B and 6D. Common bunt resistance QTL in overlapping or neighbouring locations to *QBt.ifa-1B* were also identified by Fofana et al. (2008), Dumalasová et al. (2012) and Muellner et al. (2021). In all three studies, QTL on other chromosomes were also detected but the 1B-locus had the largest effect on CBI levels. As *QBt.ifa-1B* was only detected in MP-PR2 but not in any other MP with PI 166910 as the resistant parent, we conclude that it is an additional resistance factor present only in this specific subline used as parent in this cross, but not for MP-PR1 or MP-PL.

#### Additional QTL

*QBt.ifa-2A* was the second largest effect QTL in MP-MM and located at the very proximal end of the chromosome arm. Bokore et al. (2019) found markers on chromosome 2A associated with CBI, but their QTL was located at 746 Mbp and is therefore not corresponding to the region we identified. To our knowledge, no other study investigating common bunt resistance has detected QTL on chromosome 2A, so we conclude that *QBt.ifa-2A* represents a new bunt resistance source, possibly specific to M822123.

A minor effect QTL close to the centromere of chromosome 7B was detected by Dumalasová et al. (2012) using SSR markers. Blasting the publicly available markers against the latest wheat reference sequence yielded physical positions between 417 and 544 Mbp on chromosome 7B, though, leading to the conclusion that the QTL detected by Dumalasová et al. (2012) is not corresponding to *QBt.ifa-7B*. Mourad et al. (2018) detected a significant marker-trait-association between common bunt resistance and a SNP at 18.1 Mbp on chromosome 7B in their genome-wide association study. This marker had an allele effect of $-$0.17 and its position is overlapping with the QTL interval for *QBt.ifa-7B*. Both Dumalasová et al. (2012) and Mourad et al. (2018) report that none of the known bunt resistance genes has been mapped to the short arm of chromosome 7B and this is still true in 2023 according to our literature research.

Loci conferring bunt resistance in regions corresponding to *QBt.ifa-1A* have been identified in previous studies. Ehn et al. (2022) found significant marker-trait associations of two SNPs at 473.97 Mbp on chromosome 1A with common bunt incidence in a GWA study conducted on a diversity panel. Muellner et al. (2021) investigated both common and dwarf bunt resistance in their study and identified a QTL at 380.97–516.67 Mbp (based on IWGSC RefSeq v1.0 (Appels et al. 2018)) on chromosome 1A which was effective against both diseases. A locus conferring dwarf bunt resistance in this region was also mapped by Chen et al. (2016), indicating that *QBt.ifa-1A*, despite its comparably small effect in MP-PR2, may contribute to resistance against both bunt diseases.

## Conclusion

The Turkish landrace PI 166910 has been described as a source of efficient bunt resistance which is only overcome by very few of the currently known isolates (Goates and Bockelman 2012). Goates (2012) lists only two isolates of dwarf bunt that show virulence against the resistance factor of PI 166910, *Bt11*, in his experiments. We confirm the high and stable resistance of this wheat accession in our study. Large proportions of lines in all MPs showed complete resistance in trials across six years in total and against two different local common bunt inocula. A QTL on the long arm of chromosome 6D designated *QBt.ifa-6DL* was identified in all MPs and all data sets. It had a consistently significant and decreasing effect on CBI and showed epistatic interactions with additional QTL on other chromosomes. The obtained data combined suggest that *QBt.ifa-6DL* corresponds to the bunt resistance factor *Bt11* postulated to be present in the resistant crossing partners of our MPs. Based on the evidence we collected, *QBt.ifa-6DL* is likely different from *Bt9* mapped by Steffan et al. (2017) and Wang et al. (2019), although a final proof is not yet possible due to sparse marker polymorphisms on the distal end of 6DL. The provided lines and SNP markers between 492.6 and 495.2 Mbp on chromosome 6D pave the way to deploy the promising allele *QBt.ifa-6DL*—*Bt11* in bunt resistance breeding through marker assisted selection. PI 166910 inherits additional resistance loci, notably *QBt.ifa-4BS* and *QBt.ifa-1BS*, which contribute to the high and robust bunt resistance response of this accession and its descendants.

## Supplementary Information

Below is the link to the electronic supplementary material.

Supplementary file 1 (xlsx 572 KB)

Supplementary file 2 (pdf 187 KB)

Supplementary file 3 (pdf 162 KB)

Supplementary file 4 (pdf 63 KB)

Supplementary file 5 (csv 27920 KB)

Supplementary file 6 (csv 1347 KB)

## Data Availability

All data generated during this study are included in this published article and its supplementary information files.

## References

[CR1] Abdalla O (1984) Inheritance of resistance in some wheat introductions to selected races of bunt, Tilletia caries (DC) Tul. and Tilletia foetida (Wallr) Liro (Ph. D. thesis). Oregon State University

[CR2] AGES (2022) Österreichische Beschreibende Sortenliste 2022 Landwirtschaftliche Pflanzenarten. AGES Austrian Agency for Health and Food Safety GmbH. https://bsl.baes.gv.at/fileadmin/BSL/pdfVersion/BSL2022.pdf (Schriftenreihe 21/2022). Accessed 29 Apr 2023

[CR3] Alaux M, Rogers J, Letellier T, Flores R, Alfama F, Pommier C, Quesneville H (2018). Linking the international wheat genome sequencing consortium bread wheat reference genome sequence to wheat genetic and phenomic data. Genome Biol.

[CR4] Bengtsson T, Novakazi F, Alamrani M, Edin E, Andersson B, Henriksson T, Berlin A (2023) The stinking comeback - measures to understand the cause of the re-emergence of common bunt in Swedish winter wheat? Institute of Biotechnology in Plant Production & Institute of Plant Breeding at BOKU Vienna (Eds.), XXII International Workshop on Bunt and Smut Diseases. https://boku.ac.at/fileadmin/data/H03000/H97000/H97100/pdf/Book_of_Abstracts_Bunt_and_Smut_Workshop_2023.pdf. Accessed 24 sept 2023

[CR5] Bhatta M, Baenziger P, Waters B, Poudel R, Belamkar V, Poland J, Morgounov A (2018). Genome-wide association study reveals novel genomic regions associated with 10 grain minerals in synthetic hexaploid wheat. Int J Mol Sci.

[CR6] Blazkova V, Bartos P (2002). Virulence pattern of European bunt samples (Tilletia tritici and T.laevis) and sources of resistance. Cereal Res Commun.

[CR7] Bokore FE, Cuthbert RD, Knox RE, Singh A, Campbell HL, Pozniak CJ, Ruan Y (2019). Mapping quantitative trait loci associated with common bunt resistance in a spring wheat (triticum aestivum l.) variety lillian. Theor Appl Genet.

[CR8] Borgen A (2000). Perennial survival of common bunt (Tilletia tritici) in soil under modern farming practice. J Plant Dis Prot.

[CR9] Borgen A, Davanlou M (2000). Biological contol of common bunt (Tilletia tritici). J Crop Prod.

[CR10] Broman K, Sen S (2011). A guide to qtl mapping with r/qtl (Statistics for Biology and Health).

[CR11] Broman K, Wu H, Sen S, Churchill G (2003). R/qtl: QTL mapping in experimental crosses. Bioinformatics.

[CR12] Bruehl G (1990) Cereal research at Pullman. History of the Department of Plant Pathology. https://plantpath.wsu.edu/wpcontent/uploads/sites/2193/2017/11/History.pdf

[CR13] Cadot V, Orgeur G, Baldwin T, Gombert J, Fontaine L, Du Cheyron PP, Grimault VV (2021). Carie abble - carie commune : étude de la variabilité des populations en france et développement d’un test de résistance variétale pour l’inscription des variétés de blé tendre en agriculture biologique. Innov Agrono.

[CR14] Chen J, Guttieri MJ, Zhang J, Hole D, Souza E, Goates B (2016). A novel QTL associated with dwarf bunt resistance in Idaho 444 winter wheat. Theor Appl Genet.

[CR15] Chen J, Wheeler J, Zhao W, Klassen N, O’Brien K, Marshall JM, Chen X (2018). Registration of ‘UI Sparrow’ Wheat. J Plant Regist.

[CR16] Cherewick W (1953). Smut diseases of cultivated plants in Canada.

[CR17] Dumalasová V (2021) Reaction of Czech registered varieties and sources of resistance to common bunt and dwarf bunt. Institute of Biotechnology in Plant Production & Institute of Plant Breeding at BOKU Vienna. XXI International Workshop on Bunt and Smut Diseases. from https://bunt.boku.ac.at/wpcontent/uploads/2021/05/Book-of-Abstracts.pdf. Accessed apr 2023

[CR19] Dumalasová V, Simmonds J, Bartoš P, Snape J (2012). Location of genes for common bunt resistance in the European winter wheat cv. Trintella. Euphytica.

[CR20] Ehn M, Michel S, Morales L, Gordon T, Dallinger H, Buerstmayr H (2022). Genome-wide association mapping identifies common bunt (Tilletia caries) resistance loci in bread wheat (Triticum aestivum) accessions of the USDA National Small Grains Collection. Theor Appl Genet.

[CR21] Flor H (1956). The complementary genic systems in flax and flax rust. Adv Genet.

[CR22] Fofana B, Humphreys DG, Cloutier S, McCartney CA, Somers DJ (2008). Mapping quantitative trait loci controlling common bunt resistance in a doubled haploid population derived from the spring wheat cross RL4452 x AC Domain. Mol Breed.

[CR24] Goates B, Wilcoxson R, Saari E, Ballantyne B (1996). Common bunt and dwarf bunt. Bunt and smut diseases of wheat: concepts and methods of disease management.

[CR25] Goates B (2004) Resistance genes and sources of resistance for control of dwarf bunt of wheat (TCK) caused by Tilletia controversa (Kuhn). Phytopathology, In: 15th International Plant Protection Congress, Beijing, China

[CR26] Goates BJ (2012). Identification of new pathogenic races of common bunt and dwarf bunt fungi and evaluation of known races using an expanded set of differential wheat lines. Plant Dis.

[CR27] Goates BJ, Bockelman HE (2012). Identification of new sources of high levels of resistance to dwarf bunt and common bunt among winter wheat landraces in the usda-ars national small grains collection. Crop Sci.

[CR28] Gogna A, Schulthess A, Röder M, Ganal M, Reif J (2022). Gabi wheat a panel of European elite lines as central stock for wheat genetic research. Sci Data.

[CR29] Gordon T, Wang R, Hole D, Bockelman H, Michael Bonman J, Chen J (2020). Genetic characterization and genome-wide association mapping for dwarf bunt resistance in bread wheat accessions from the USDA National Small Grains Collection. Theor Appl Genet.

[CR30] Haley C, Knott S (1992). A simple regression method for mapping quantitative trait loci in line crosses using flanking markers. Heredity.

[CR31] Harlan J (1950). Collection of crop plants in Turkey, 1948. Agron J.

[CR32] Hoffman J (1982). Bunt of wheat. Plant Dis.

[CR33] Hoffman J, Metzger R (1976). Current status of virulence genes and pathogenic races of the wheat bunt fungi in the Northwestern USA. Phytopathology.

[CR34] Hole D, Clawson S, Young S, Roche D (2002). Registration of ‘Golden Spike’ wheat. Crop Sci.

[CR36] Johnsson L (1992). Climate factors influencing attack of common bunt (Tilletia caries (DC) Tul.) in winter wheat in 1940–1988 in Sweden. J Plant Dis Prot.

[CR37] Joshi P, Dhillon G, Hole D, Gordon T, Buerstmayr H, Ehn M, Chen J (2023) Assessment of Common Bunt and Dwarf Bunt Resistance in Bt-Differential Lines Grown in Diverse Environments. In: Institute of Biotechnology in Plant Production & Institute of Plant Breeding at BOKU Vienna, XXII International Workshop on Bunt and Smut Diseases. https://boku.ac.at/fileadmin/data/H03000/H97000/H97100/pdf/Book_of_Abstracts_Bunt_and_Smut_Workshop_2023.pdf. Accessed on 24 July 2023

[CR39] Kuepper G (2010) A Brief Overview of the History and Philosophy of Organic Agriculture. Kerr Center for Sustainable Agriculture, Poteau. https://kerrcenter.com/wpcontent/uploads/2014/08/organic-philosophy-report.pdf. Accessed on 04 May 2023

[CR40] Laroche A, Demeke T, Gaudet D, Puchalski B, Frick M, McKenzie R (2000). Development of a PCR marker for rapid identification of the Bt-10 gene for common bunt resistance in wheat. Genome.

[CR41] Liatukas Z, Ruzgas V (2008). Resistance genes and sources for the control of wheat common bunt (Tilletia tritici (DC.) Tul.). Biologija.

[CR42] Line R (1993). Integrated pest management for wheat: IPM in a wide-ranging system. Plant Dis.

[CR43] Manichaikul A, Moon JY, Sen S, Yandell BS, Broman KW (2009). A model selection approach for the identification of quantitative trait loci in experimental crosses. Allowing Epistasis. Genetics.

[CR44] Martens J, Seaman W, Atkinson T (1984). Diseases of field crops in Canada.

[CR45] Matanguihan J, Murphy KM, Jones SS (2011). Identification of pathogenic races and microsatellite markers of Tilletia caries (D.C.) Tul. & C. Tul. and mapping of a common bunt resistance gene in winter wheat. Plant Dis.

[CR46] Menzies J, Knox R, Popovic Z, Procunier J (2006). Common bunt resistance gene Bt10 located on wheat chromosome 6D. Can J Plant Sci.

[CR47] Metzger R, Hoffman J (1978). New races of common bunt useful to determine resistance of wheat to dwarf bunt. Crop Sci.

[CR48] Mourad AMI, Sallam A, Belamkar V, Mahdy E, Bakheit B, Abo El-Wafaa A, Stephen Baenziger P (2018). Genetic architecture of common bunt resistance in winter wheat using genome-wide association study. BMC Plant Biol.

[CR49] Muellner AE, Buerstmayr M, Eshonkulov B, Hole D, Michel S, Hagenguth JF, Buerstmayr H (2021). Comparative mapping and validation of multiple disease resistance QTL for simultaneously controlling common and dwarf bunt in bread wheat. Theor Appl Genet.

[CR50] Muellner AE, Eshonkulov B, Hagenguth J, Pachler B, Michel S, Buerstmayr M, Buerstmayr H (2020). Genetic mapping of the common and dwarf bunt resistance gene Bt12 descending from the wheat landrace PI119333. Euphytica.

[CR51] Muñoz F, Sanchez L (2020) breedr: statistical methods for forest genetic resources analysts [Computer software manual]. https://github.com/famuvie/breedR (R package version 0.12-5). Accessed on 29 Apr 2023

[CR52] Ouellette L, Reid R, Blanchard S, Brouwer C (2018). LinkageMapView—rendering high resolution linkage and QTL maps. Bioinformatics.

[CR53] R Core Team (2021) R: A Language and Environment for Statistical Computing [Computer software manual]. Vienna, Austria. https://www.R-project.org/. Accessed on 29 Apr 2023

[CR54] Ritzer E, Ehn M, Oberforster M, Buerstmayr H (2022) Pathogenicity patterns of Austrian Tilletia caries isolates on winter wheat Triticum aestivum. Saatgut Austria In: 72nd Plant Breeders Conference, (pp 75-76). 10.5281/zenodo.5667799

[CR55] RWA AG (2023) Tilliko. https://www.diesaat.at/wp-content/uploads/2020/02/sortenpasswinterweizen-tilliko-bio.pdf (Raiffeisen Ware Austria). Accessed on 04 May 2023

[CR56] Saari E, Mamluk O, Wilcoxon R (1996). Bunt and smut diseases of wheat: concepts and methods of disease management. Chap, wheat bunts and smuts.

[CR57] Saghai-Maroof M, Soliman K, Jorgensen R, Allard R (1984). Ribosomal DNA spacer-length polymorphisms in barley: mendelian inheritance, chromosomal location, and population dynamics. Proc Natl Acad Sciences USA.

[CR58] Sen S, Churchill GA (2001). A Statistical Framework for Quantitative Trait Mapping. Genetics.

[CR59] Singh A, Knox RE, DePauw RM, Singh AK, Cuthbert RD, Kumar S, Campbell HL (2015). Genetic mapping of common bunt resistance and plant height QTL in wheat. Theor Appl Genet.

[CR60] Souza E, Guttieri M, Mclean R (2004). Registration of ‘moreland’ wheat. Crop Sci.

[CR61] Steffan PM, Torp AM, Borgen A, Backes G, Rasmussen SK (2017). Mapping of common bunt resistance gene Bt9 in wheat. Theor Appl Genet.

[CR62] Strube H (1967) Merkmalskorrelationen bei Hybridmais und ihre Bedeutung fur die Selektion (Ph.D. thesis). Universität Hohenheim, Schloss Hohenheim 1, 70599 Stuttgart

[CR63] Taylor J, Butler D (2017). R package ASMap: efficient genetic linkage map construction and diagnosis. J Stat Softw.

[CR64] Appels R, Eversole K, Stein N, Feuillet C, Keller B, Wang L (2018). Shifting the limits in wheat research and breeding using a fully annotated reference genome. Int Wheat Genom Seq Consort Sci.

[CR67] Wang R, Gordon T, Hole D, Zhao W, Isham K, Bonman JM, Chen J (2019). Identification and assessment of two major QTLs for dwarf bunt resistance in winter wheat line ‘IDO835’. Theor Appl Genet.

[CR66] Walkowiak S, Gao L, Haberer G, Kassa MT, Brinton J, Kolodziej MC, Pozniak CJ (2020). Multiple wheat genomes reveal global variation in modern breeding. Nature.

[CR69] Yao E, Blake VC, Cooper L, Wight CP, Michel S, Cagirici HB, Sen TZ (2022). GrainGenes: a data-rich repository for small grains genetics and genomics. Database.

[CR70] Zhu T, Wang L, Rimbert H, Rodriguez JC, Deal KR, De Oliveira R, Luo M-C (2021). Optical maps refine the bread wheat Triticum aestivum cv. Chinese Spring genome assembly. Plant J.

