# Supplementary material for: Wheat (Triticum aestivum) chromosome 6D harbours the broad spectrum common bunt resistance gene Bt11
Source: Theor Appl Genet. 2023 Sep 7;136(9):207. doi: 10.1007/s00122-023-04452-5 (PMC10485103; doi:10.1007/s00122-023-04452-5)
Supplement: Supplementary file 2 — (pdf 187 KB) [file 122_2023_4452_MOESM2_ESM.pdf]

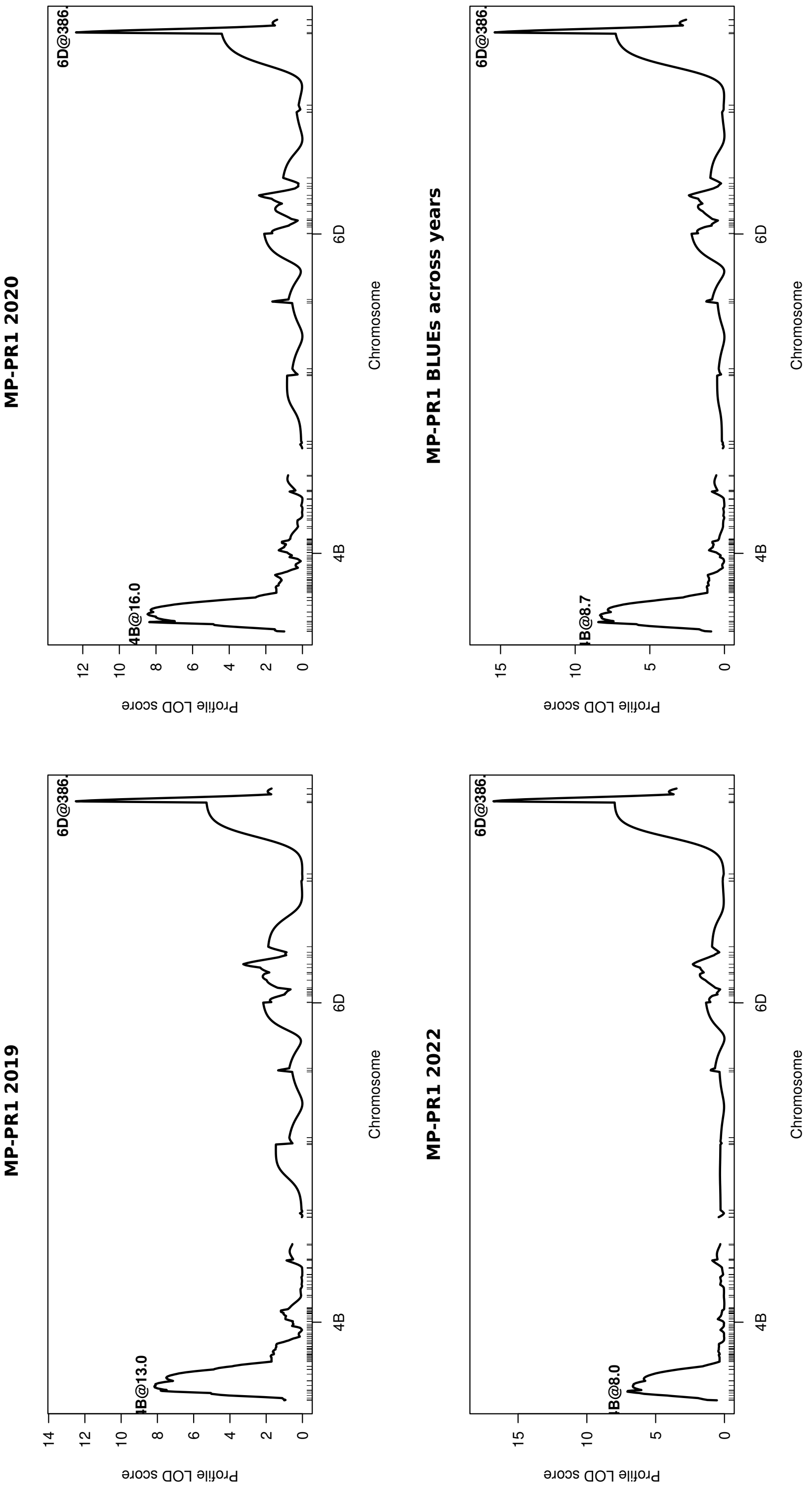

(a) LOD-profiles in MP-PR1

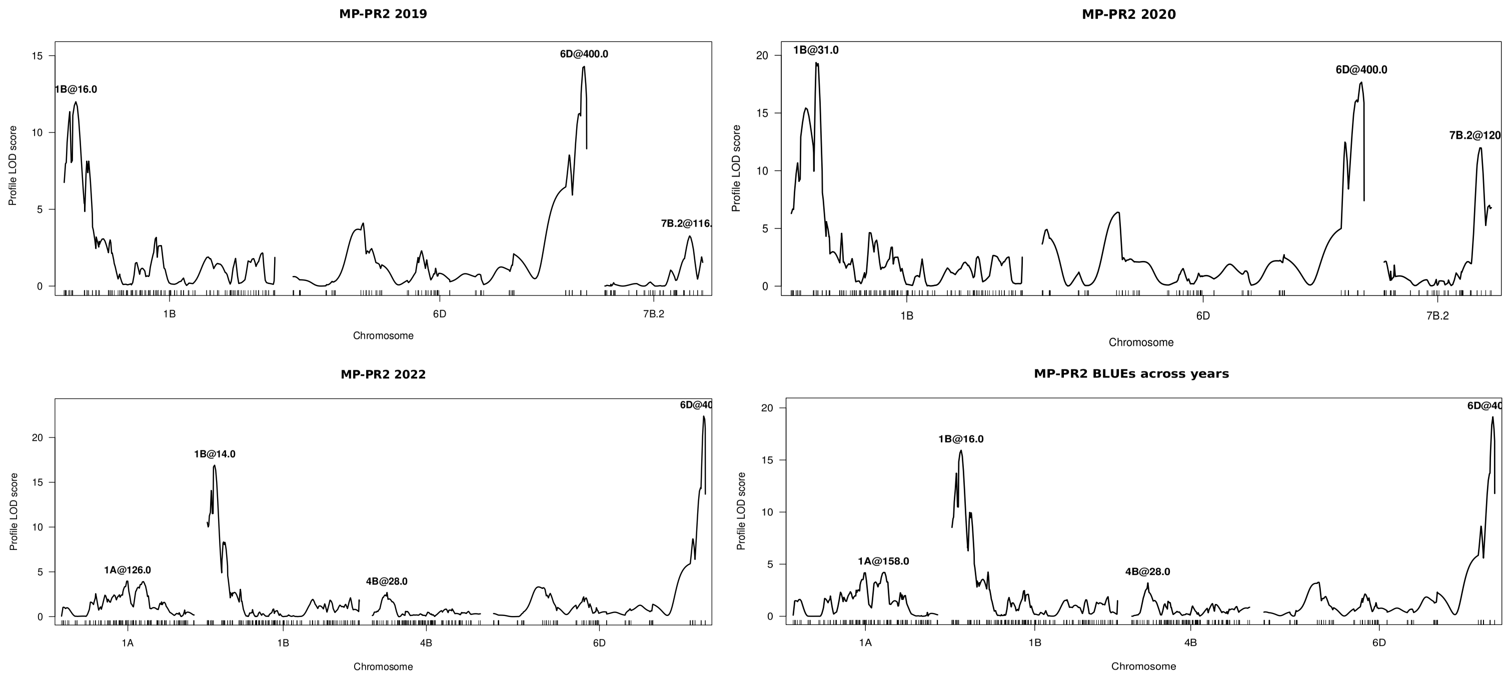

(b) LOD-profiles in MP-PR2

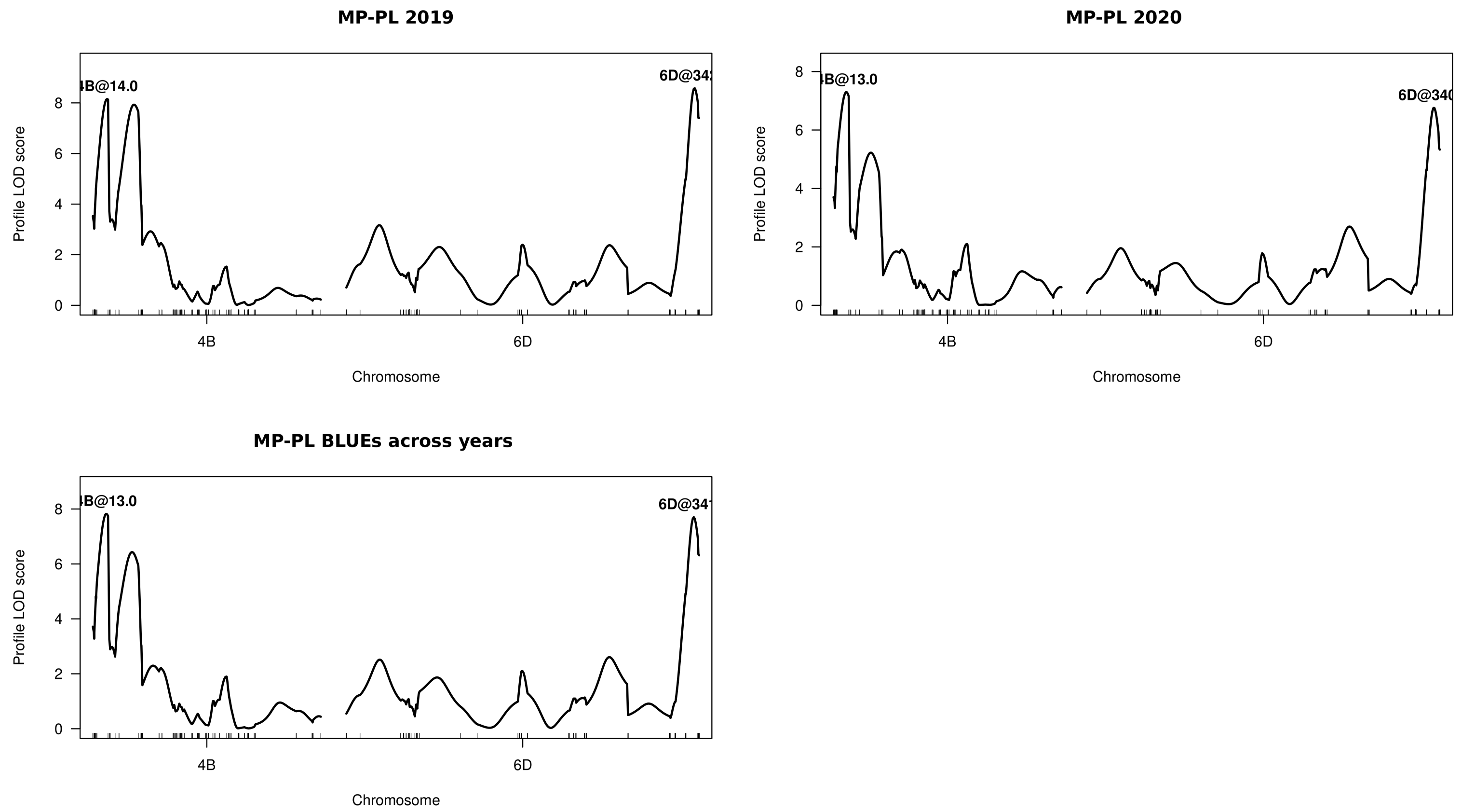

(c) LOD-profiles in MP-PL

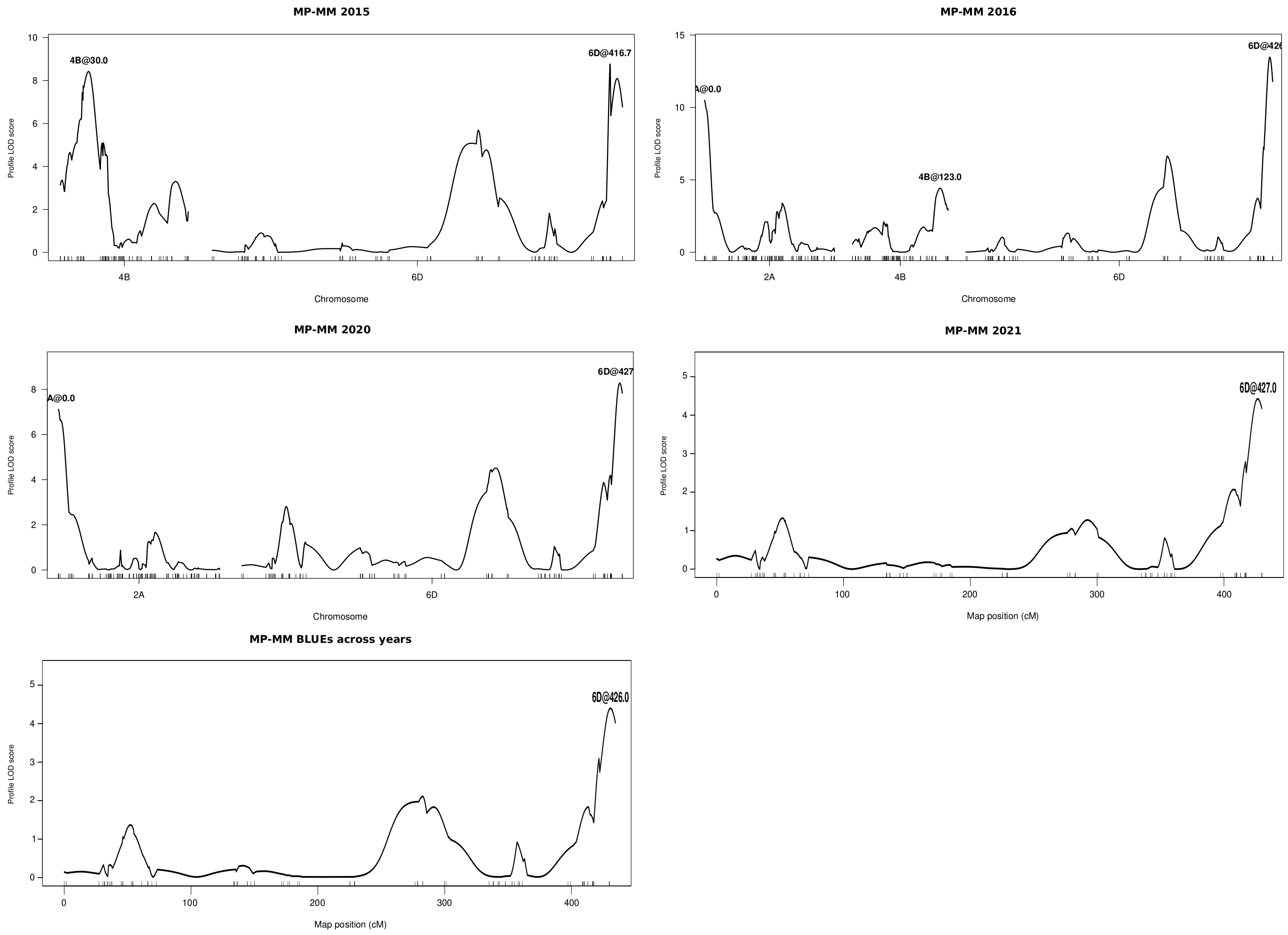

(d) LOD-profiles in MP-MM

Supplementary File 6: LOD profiles based on model selection results from the *stepwise*-function in R package *r/qtl* (Broman et al., 2003). Positions of LOD-peaks are indicated in centi Morgan (cM). LOD-profiles are shown for common bunt incidence scores in each individual experiment and BLUEs across experiments for each mapping populations (MP) separately: (a) MP-PR1 = PI 166910 × 'Rainer' (b) MP-PR2 = 'Rainer' × PI 166910 (c) MP-PL = PI 166910 × 'Lukullus' (d) MP-MM = M822123 × 'Mulan'
