# Supplementary material for: Wheat (Triticum aestivum) chromosome 6D harbours the broad spectrum common bunt resistance gene Bt11
Source: Theor Appl Genet. 2023 Sep 7;136(9):207. doi: 10.1007/s00122-023-04452-5 (PMC10485103; doi:10.1007/s00122-023-04452-5)
Supplement: Supplementary file 3 — (pdf 162 KB) [file 122_2023_4452_MOESM3_ESM.pdf]

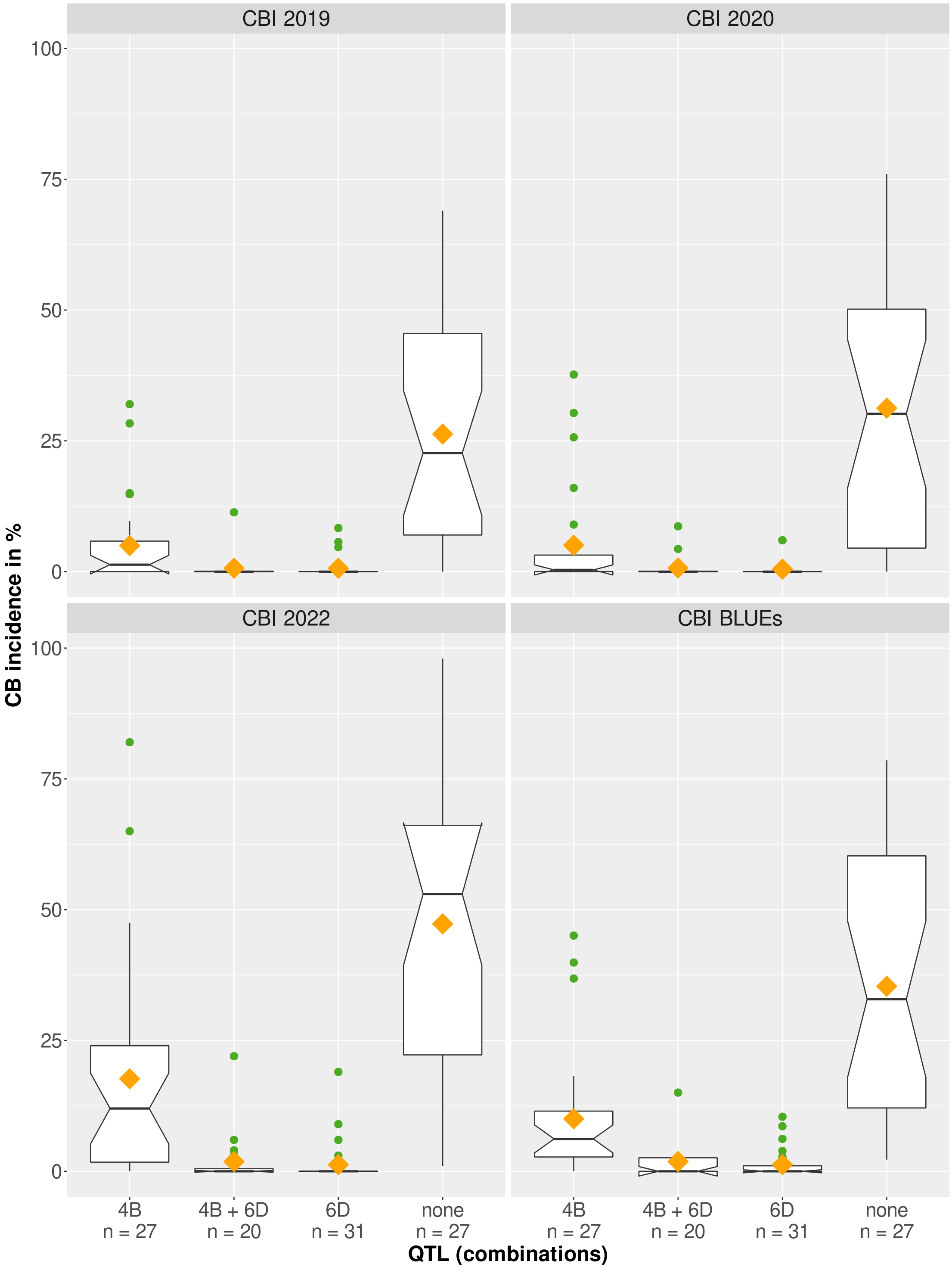
CBI 2019
CBI 2020
CBI 2022
CBI BLUEs
CB incidence in %
100
75
50
25
0
4B
n = 27
4B + 6D
n = 20
6D
n = 31
none
n = 27
QTL (combinations)

(a) MP-PR1

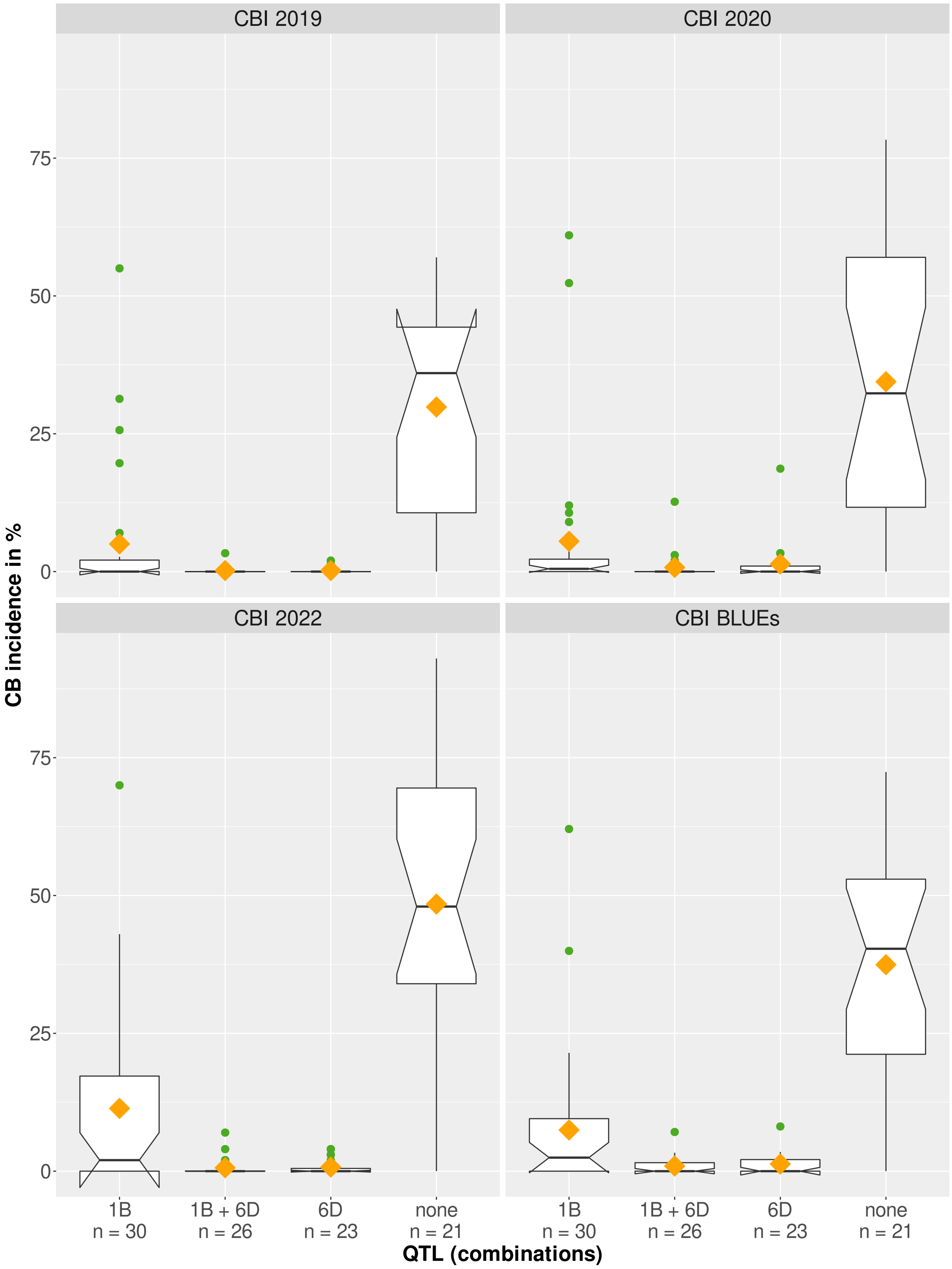
CBI 2019
CBI 2020
CBI 2022
CBI BLUEs
CB incidence in %
0
25
50
75
1B
n = 30
1B + 6D
n = 26
6D
n = 23
none
n = 21
QTL (combinations)

(b) MP-PR2

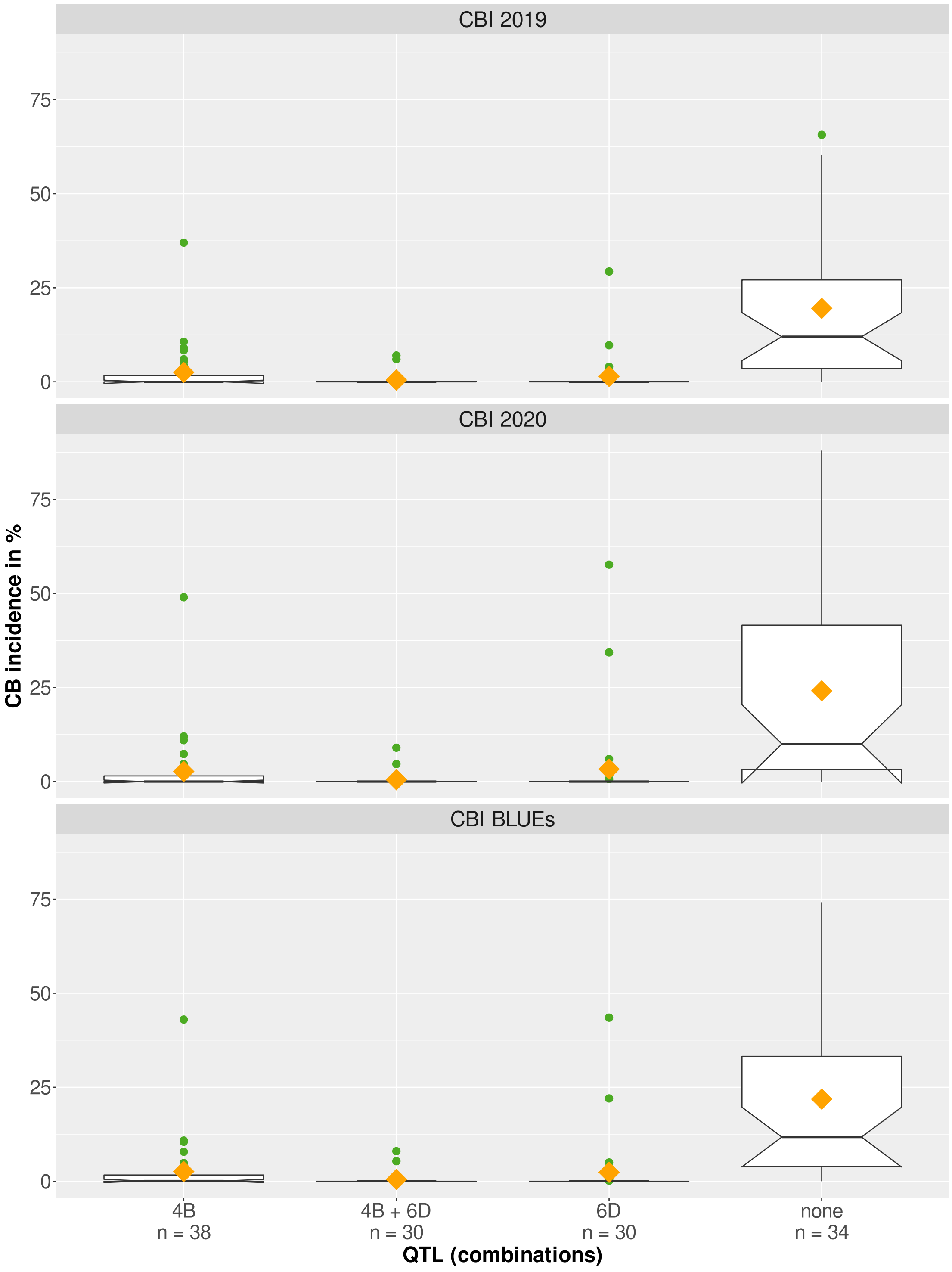
CBI 2019
CBI 2020
CBI BLUEs
CB incidence in %
0
25
50
75
4B
n = 38
4B + 6D
n = 30
6D
n = 30
none
n = 34
QTL (combinations)

(c) MP-PL

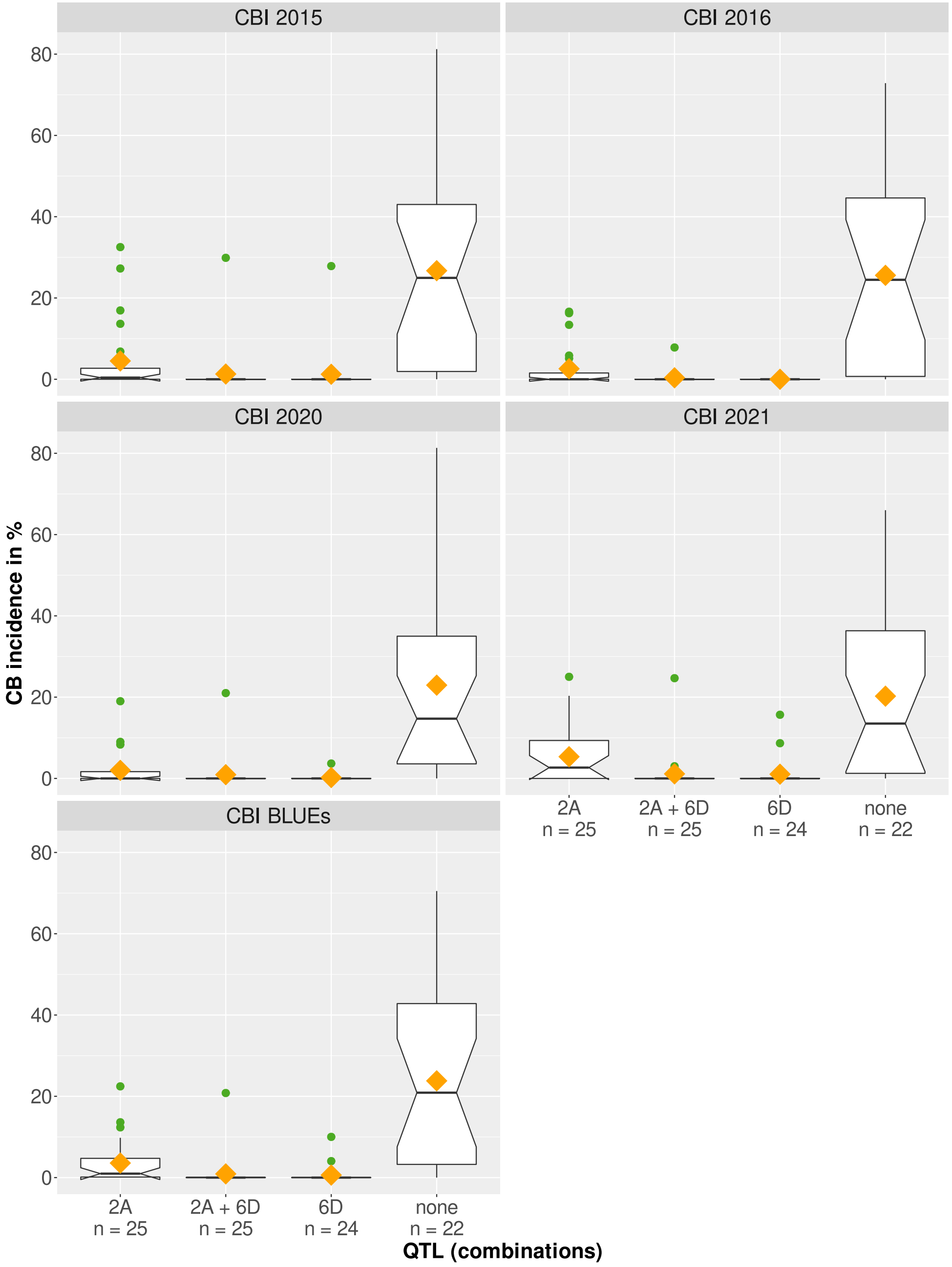

(d) MP-MM

Supplementary File 7: Boxplots showing common bunt incidence (CBI) levels in percent for recombinant inbred lines (RILs) harbouring resistance conferring alleles for different QTL(-combinations). Results are shown for all four mapping populations (MPs) individually: (a) 'Rainer' × PI 166910 (b) PI 166910 × 'Rainer' (c) PI 166910 × 'Lukullus' (d) M822123 × 'Mulan'. Orange squares mark average CBI, green dots indicate outliers. The number of RILs harbouring the respective QTL(-combinations) is given on the x-axis
