# Supplementary material for: Wheat (Triticum aestivum) chromosome 6D harbours the broad spectrum common bunt resistance gene Bt11
Source: Theor Appl Genet. 2023 Sep 7;136(9):207. doi: 10.1007/s00122-023-04452-5 (PMC10485103; doi:10.1007/s00122-023-04452-5)
Supplement: Supplementary file 4 — (pdf 63 KB) [file 122_2023_4452_MOESM4_ESM.pdf]

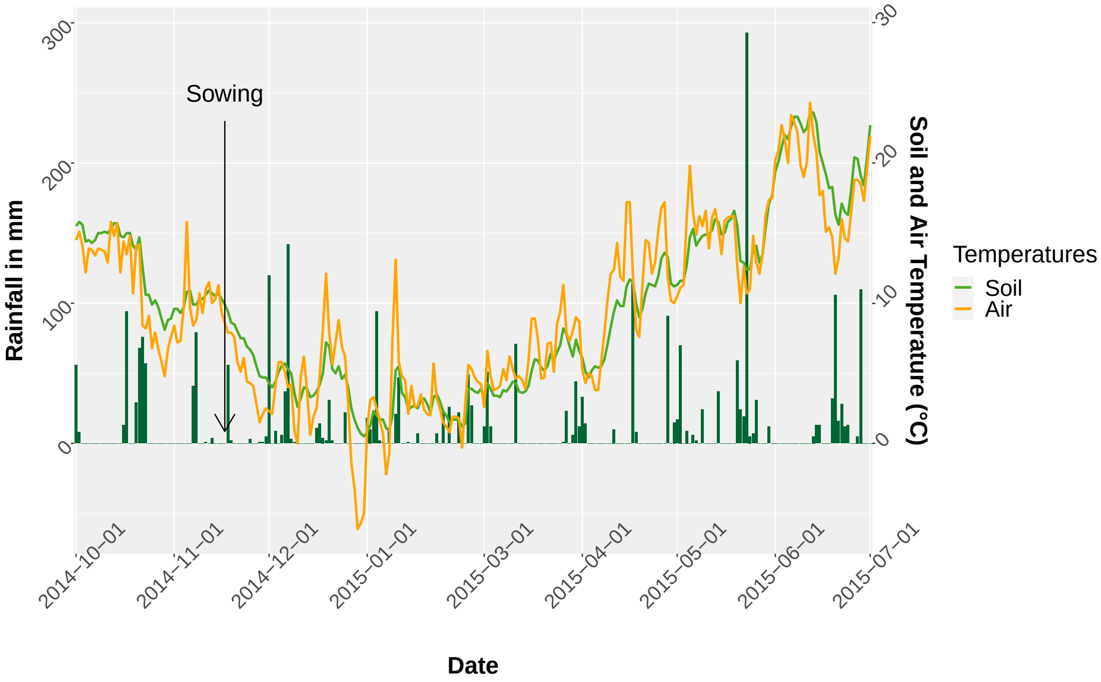

(a) season 2014/15

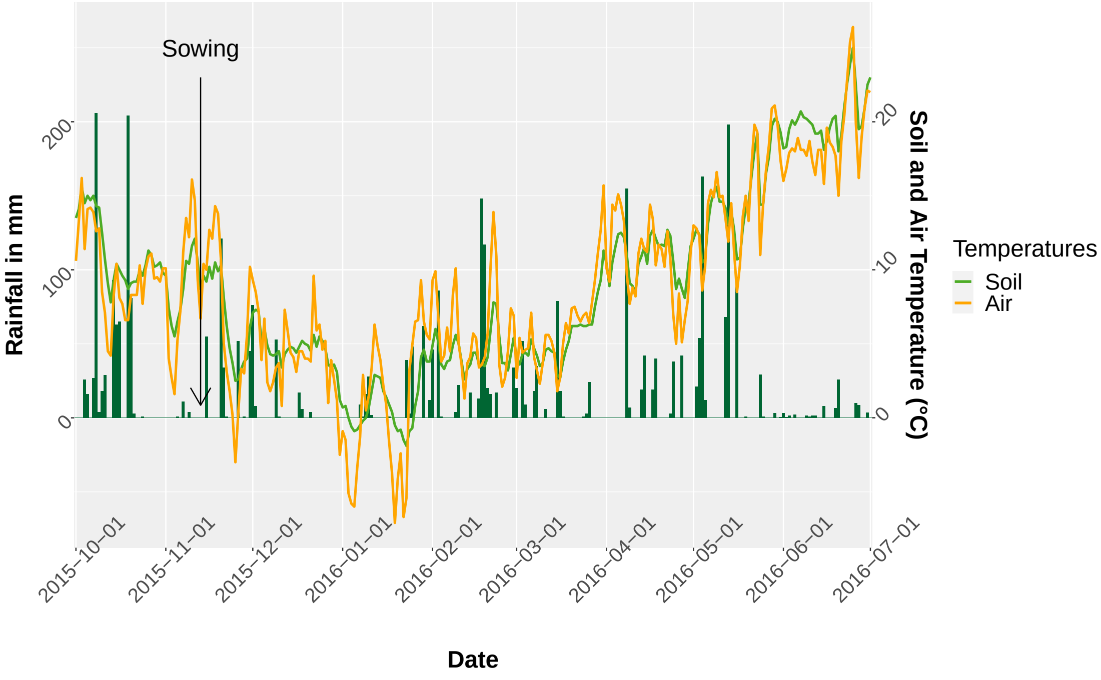

(b) season 2015/16

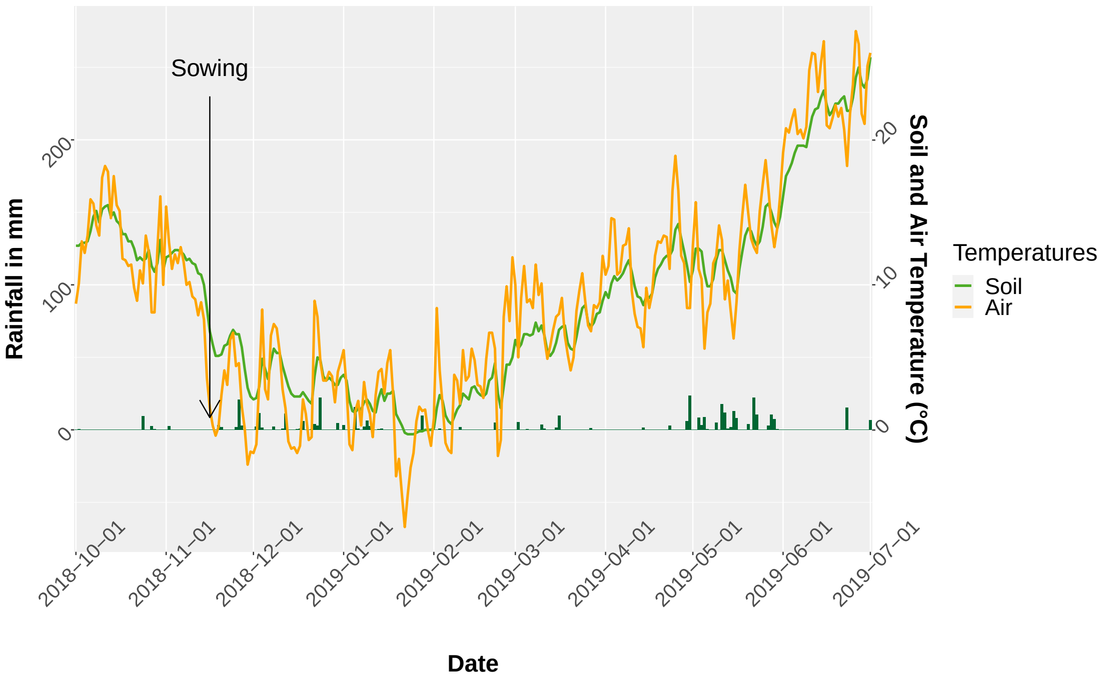

(c) season 2018/19

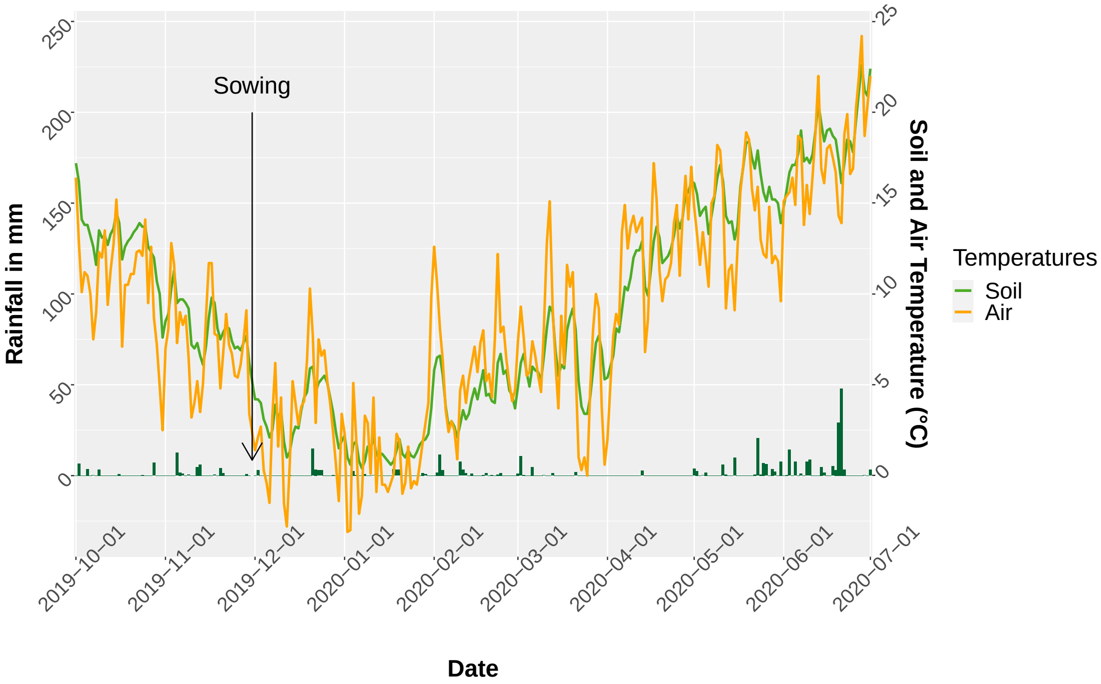

(d) season 2019/20

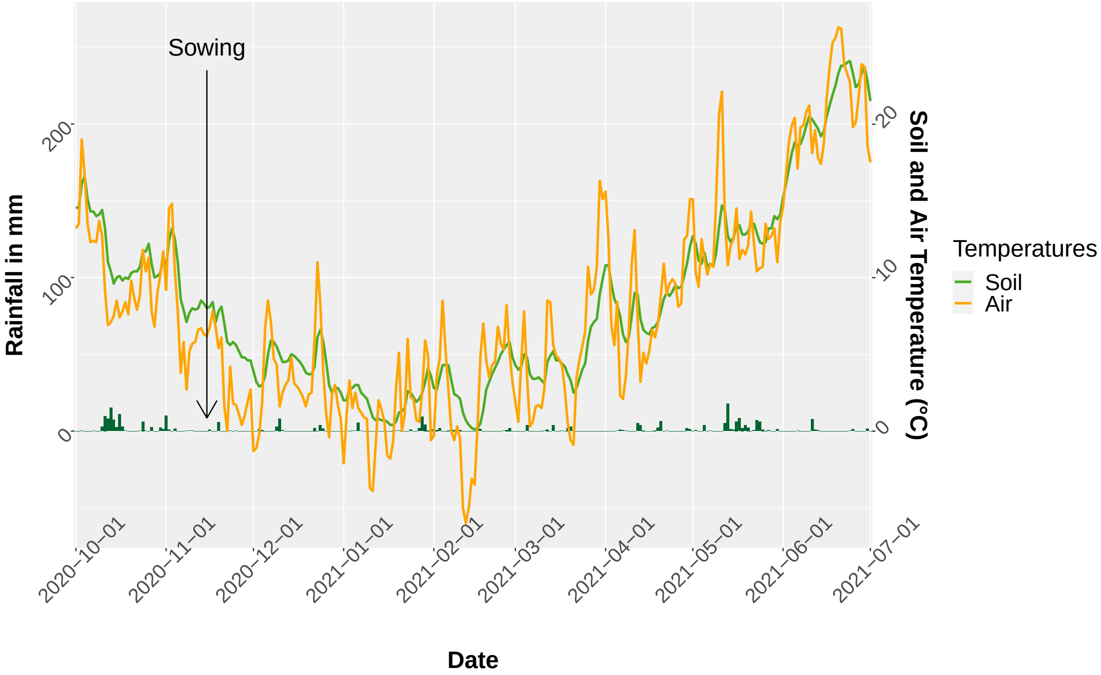

(e) season 2020/21

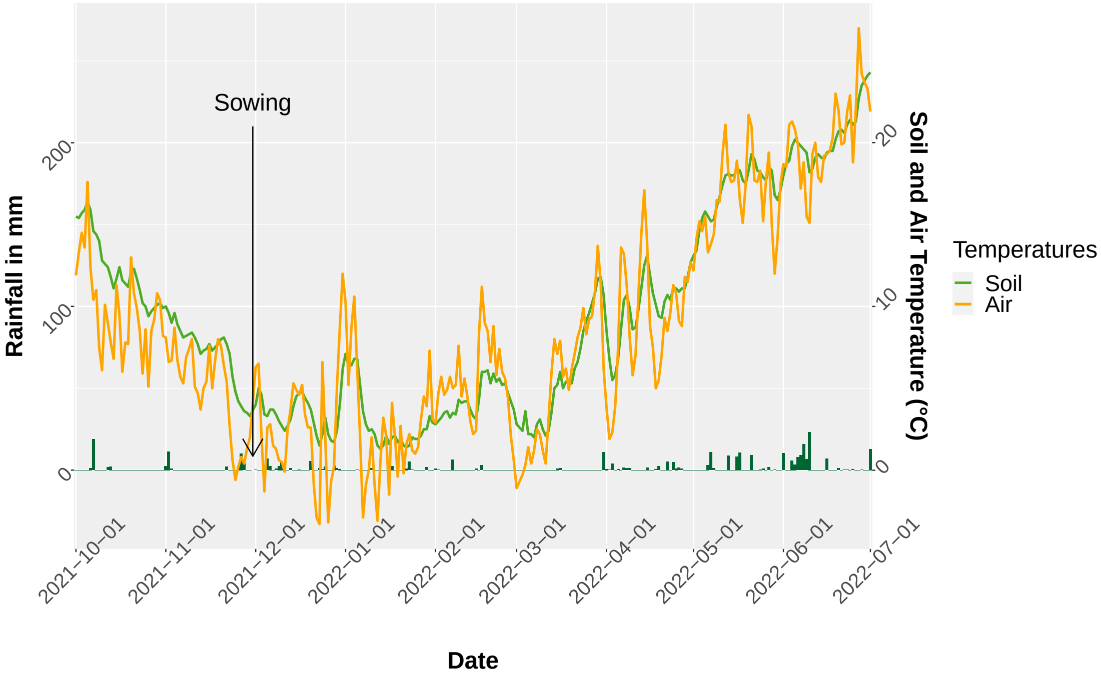

(f) season 2021/22

Supplementary File 8: Soil (green curve) and air (orange curve) temperature in degrees Celsius as well as rainfall in mm (dark green bars) for all growing seasons in which common bunt field trials for this study were conducted. The sowing date is indicated with an arrow
